# Changes in rapid HIV treatment initiation after national “treat all” policy adoption in 6 sub-Saharan African countries: Regression discontinuity analysis

**DOI:** 10.1371/journal.pmed.1002822

**Published:** 2019-06-10

**Authors:** Olga Tymejczyk, Ellen Brazier, Constantin T. Yiannoutsos, Michael Vinikoor, Monique van Lettow, Fred Nalugoda, Mark Urassa, Jean d’Amour Sinayobye, Peter F. Rebeiro, Kara Wools-Kaloustian, Mary-Ann Davies, Elizabeth Zaniewski, Nanina Anderegg, Grace Liu, Nathan Ford, Denis Nash, on behalf of the IeDEA consortium

**Affiliations:** 1 Institute for Implementation Science in Population Health, City University of New York, New York, New York, United States of America; 2 Graduate School of Public Health and Health Policy, City University of New York, New York, New York, United States of America; 3 Richard M. Fairbanks School of Public Health, Indiana University, Indianapolis, Indiana, United States of America; 4 Centre for Infectious Disease Research in Zambia, Lusaka, Zambia; 5 Department of Medicine, University of Alabama, Birmingham, Alabama, United States of America; 6 Dignitas International, Zomba, Malawi; 7 Dalla Lana School of Public Health, University of Toronto, Toronto, Ontario, Canada; 8 Rakai Health Sciences Program, Kalisizo and Entebbe, Uganda; 9 Mwanza Intervention Trials Unit, National Institute for Medical Research, Mwanza, Tanzania; 10 Rwanda Military Hospital, Kigali, Rwanda; 11 Vanderbilt University School of Medicine, Nashville, Tennessee, United States of America; 12 Division of Infectious Diseases, Indiana University School of Medicine, Indianapolis, Indiana, United States of America; 13 Centre for Infectious Disease Epidemiology and Research, School of Public Health and Family Medicine, University of Cape Town, Cape Town, South Africa; 14 Institute of Social and Preventive Medicine, University of Bern, Bern, Switzerland; 15 Global Hepatitis Programme, HIV/AIDS Department, World Health Organization, Geneva, Switzerland; University of Southampton, UNITED KINGDOM

## Abstract

**Background:**

Most countries have formally adopted the World Health Organization’s 2015 recommendation of universal HIV treatment (“treat all”). However, there are few rigorous assessments of the real-world impact of treat all policies on antiretroviral treatment (ART) uptake across different contexts.

**Methods and findings:**

We used longitudinal data for 814,603 patients enrolling in HIV care between 1 January 2004 and 10 July 2018 in 6 countries participating in the global International epidemiology Databases to Evaluate AIDS (IeDEA) consortium: Burundi (*N =* 11,176), Kenya (*N =* 179,941), Malawi (*N =* 84,558), Rwanda (*N =* 17,396), Uganda (*N =* 96,286), and Zambia (*N =* 425,246). Using a quasi-experimental regression discontinuity design, we assessed the change in the proportion initiating ART within 30 days of enrollment in HIV care (rapid ART initiation) after country-level adoption of the treat all policy. A modified Poisson model was used to identify factors associated with failure to initiate ART rapidly under treat all. In each of the 6 countries, over 60% of included patients were female, and median age at enrollment ranged from 32 to 36 years. In all countries studied, national adoption of treat all was associated with large increases in rapid ART initiation. Significant increases in rapid ART initiation immediately after treat all policy adoption were observed in Rwanda, from 44.4% to 78.9% of patients (34.5 percentage points [pp], 95% CI 27.2 to 41.7; *p* < 0.001), Kenya (25.7 pp, 95% CI 21.8 to 29.5; *p* < 0.001), Burundi (17.7 pp, 95% CI 6.5 to 28.9; *p* = 0.002), and Malawi (12.5 pp, 95% CI 7.5 to 17.5; *p* < 0.001), while no immediate increase was observed in Zambia (0.4 pp, 95% CI −2.9 to 3.8; *p* = 0.804) and Uganda (−4.2 pp, 95% CI −9.0 to 0.7; *p* = 0.090). The rate of rapid ART initiation accelerated sharply following treat all policy adoption in Malawi, Uganda, and Zambia; slowed in Kenya; and did not change in Rwanda and Burundi. In post hoc analyses restricted to patients enrolling under treat all, young adults (16–24 years) and men were at increased risk of not rapidly initiating ART (compared to older patients and women, respectively). However, rapid ART initiation following enrollment increased for all groups as more time elapsed since treat all policy adoption. Study limitations include incomplete data on potential ART eligibility criteria, such as clinical status, pregnancy, and enrollment CD4 count, which precluded the assessment of rapid ART initiation specifically among patients known to be eligible for ART before treat all.

**Conclusions:**

Our analysis indicates that adoption of treat all policies had a strong effect on increasing rates of rapid ART initiation, and that these increases followed different trajectories across the 6 countries. Young adults and men still require additional attention to further improve rapid ART initiation.

## Abstract 2

Olga Tymejczyk and colleagues report on the impact of WHO's universal treatment recommendation for people with HIV in sub-Saharan African countries.

## Author summary

**Why was this study done?:**

- Since late 2015, the World Health Organization (WHO) has recommended that all people living with HIV (PLWH) initiate ART, regardless of disease stage and CD4 cell count, and as of mid-2018, 84% of low- and middle-income countries had formally adopted WHO’s recommendation to provide universal treatment to all PLWH.
- While modeling studies and trials have indicated that universal treatment, or “treat all,” increases rapid ART initiation and viral load suppression among patients, there is little evidence on the impact of treat all policies on uptake of ART in real-world implementation settings.

**What did the researchers do and find?:**

- We assessed rapid ART initiation (i.e., ART initiation within 30 days of enrolling into HIV care) among 814,603 patients before and after national adoption of treat all policies in 6 countries in sub-Saharan Africa (Burundi, Kenya, Malawi, Rwanda, Uganda, and Zambia).
- To compare rapid ART initiation among those enrolling into care just before and just after national adoption of the treat all policy, we used a regression discontinuity design, using the date of HIV care enrollment as a continuous eligibility assignment variable. This design creates a quasi-experimental condition where the only systematic difference between patients enrolling just before and just after treat all policy adoption is the probability of treatment eligibility.
- There were large increases in rapid ART initiation after national adoption of treat all, with 81.6% of patients initiating ART within 30 days of enrollment after the policy adoption, and little difference in rapid ART initiation across patients with different immunodeficiency status, as measured by CD4 counts.
- In Burundi, Kenya, Malawi, and Rwanda, there were large and significant increases in rapid ART initiation immediately following national adoption of treat all, ranging from 12.5- and 17.7-percentage-point increases in rapid ART initiation in Malawi and Burundi, respectively, to 25.7- and 34.5-percentage-point increases in Kenya and Rwanda.
- In Uganda and Zambia, there was no significant increase in rapid ART initiation immediately following national adoption of treat all. However, there were significant increases in the rate of rapid ART initiation in the months following treat all adoption, with each additional month following the policy adoption being associated with 2.2- and 2.6-percentage-point increases in the proportion of patients initiating ART in Uganda and Zambia, respectively.
- In the period following treat all adoption, men and young adults (aged 16–24 years) were less likely to rapidly initiate ART than women and older adults, respectively. However, for all groups, the risk of failing to rapidly initiate ART significantly decreased with elapsed time after treat all policy adoption.

**What do these findings mean?:**

- Using a regression discontinuity design and real-world service delivery data, primarily from public-sector health facilities, our findings suggest that national adoption of treat all policies has led to appreciable increases in rapid ART initiation.
- As more follow-up data become available, future research should assess longer-term outcomes, such as retention in care, viral suppression, and treatment failure among patients initiating therapy under treat all.

## Introduction

In September 2015, after a series of clinical-stage- and CD4-count-based antiretroviral therapy (ART) eligibility expansions [1], the World Health Organization (WHO) recommended that all people living with HIV (PLWH) should start ART regardless of disease stage and at any CD4 cell count [2]. In 2017, WHO recommended treatment initiation within 7 days of confirming HIV diagnosis, including same-day ART initiation when feasible [3]. This recommendation followed from the results of several randomized trials and observational studies showing that rapid ART initiation was associated with reduced mortality and morbidity, and with a greater likelihood of achieving virological suppression and retention in care [4]. If initiated rapidly, universal HIV treatment (also known as “treat all” and “test and start”) is thus an important strategy for reaching the Joint United Nations Programme on HIV/AIDS (UNAIDS) 90-90-90 targets.

Implementation of treat all is accelerating, and as of mid-2018, 84% of low- and middle-income countries had formally adopted the recommendation to provide universal treatment to PLWH [5]. Given the relatively recent rollout of treat all policies at the country level, however, there is little evidence to date on the policy’s real-world effect on ART uptake. As the number of patients on ART grows under treat all policies, it is also critical to examine how the policy impacts patient groups that have historically lagged behind in HIV care engagement (i.e., men and young adults). While changes in ART initiation following WHO’s 2009 and 2013 ART eligibility expansion recommendations have been evaluated [6–8], most estimates of the potential impact of treat all to date come from trials and modeling studies [9–13]. Although most of these research studies have reported favorable outcomes, they may not be generalizable to other contexts, including settings where health system and other resource constraints contribute to delays in policy implementation.

In this study, we used longitudinal patient data from 6 countries participating in the International epidemiology Databases to Evaluate AIDS (IeDEA) consortium to describe ART initiation rates under different country-level eligibility guidelines, to assess the impact of treat all policies on rapid ART initiation, and to identify factors associated with failure to start treatment rapidly under treat all.

## Methods

### Data sources and management

#### Patient data

IeDEA (https://www.iedea.org) captures demographic and clinical data on over 1.7 million patients receiving HIV care in 46 countries across 7 regional cohort collaborations [14]. The data represent a diverse cross-section of PLWH and HIV treatment programs, the majority of which (87%) are at public-sector health facilities, including both primary (42%) and secondary/tertiary-level sites (58%) [15]. In this analysis, we used medical records from patients enrolled in these programs between 1 January 2004 and 10 July 2018 in 6 countries in 3 regional cohorts with post-treat-all data available for analysis (Burundi and Rwanda in Central Africa, Kenya and Uganda in East Africa, and Malawi and Zambia in Southern Africa). Prior to merging and analysis, each region’s data were standardized by regional data managers in accordance with IeDEA data exchange standards for variable definitions and data formatting.

#### ART guidelines

For each country in the analysis, we identified the dates of major ART eligibility expansions to CD4 ≤ 350 cells/μl, CD4 ≤ 500 cells/μl, and treat all. We have previously described our systematic search for current and historical ART eligibility guidelines based on publicly available policy documents, published literature, and inputs from in-country experts [6]. If the exact date of ART eligibility expansion was unknown, it was assumed to have occurred on the first day of the month in which the policy was adopted. In Malawi, the date of national treat all adoption published in the country’s guidelines was adjusted to 2 months later to reflect the delays in policy rollout documented by the Ministry of Health [16].

The study utilized de-identified data approved for use by local ethical committees in each of the IeDEA regions included in the analysis.

### Inclusion criteria

Patients had to be at least 16 years old at enrollment and have at least 30 days of possible follow-up between enrollment and database closure. Patients were excluded if they transferred to an IeDEA site from another clinic or were known not to be ART naïve at enrollment (with ART defined as any regimen of at least 3 antiretroviral drugs, excluding treatment taken solely for prevention of mother-to-child transmission). We excluded sites with no patient data available for the period between care enrollment and ART initiation (i.e., pre-ART data, such as documented visits and laboratory tests prior to ART initiation), as well as sites that only reported data for ART initiators (defined as sites where fewer than 2% of patients over the study period never initiated ART).

### Outcome and exposure

The outcome of interest was rapid ART initiation, defined as initiation of treatment within 30 days of enrollment in HIV care (distinct from the 2017 WHO definition of rapid ART initiation as occurring within 7 days of HIV diagnosis) [3]. The exposure was time of enrollment in HIV care with respect to the calendar date of country-level ART eligibility expansion to treat all.

### Other definitions

To represent pre-treatment HIV disease severity, CD4 count at enrollment was defined as the CD4 measurement closest to the enrollment date within a ±90-day window, but no later than 1 week after ART initiation.

### Study design

#### Descriptive analyses

The proportion of patients initiating ART rapidly in each of the 4 ART eligibility periods (at CD4 ≤ 200 or CD4 ≤ 250 cells/μl [period 1]; at CD4 ≤ 350 cells/μl [period 2]; at CD4 ≤ 500 cells/μl [period 3]; and treat all [period 4]) was calculated and stratified by country, sex, and age group (dichotomized as 16–24 or ≥25 years to approximate the 15–24-year age category commonly used by UNAIDS) [17].

#### Effect of ART eligibility expansion to treat all on rapid ART initiation

A regression discontinuity design was applied to assess the effect of enrollment in HIV care under treat all on the proportion of patients initiating ART rapidly. This approach takes advantage of the local randomness around a cutoff-based, continuous eligibility assignment variable (in this case, calendar date of HIV care enrollment relative to the date of country-level adoption of treat all). This local randomness creates a quasi-experimental condition in which there are no systematic differences between patients enrolling in care on either side of the cutoff threshold (date of country-level treat all adoption), but patients on one side (those enrolling after treat all adoption) have a higher probability of initiating treatment than on the other, permitting a causal interpretation of observed effects [18–20]. Covariate balance tests for patients enrolling immediately before and after treat all adoption and plots of the assignment variable were completed to assess the possibility of systematic differences between patients enrolling in HIV care on either side of the threshold, as well as nonrandom enrollment before or after the treat all adoption date.

As information about each patient’s ART eligibility at enrollment (e.g., HIV stage, comorbidity, pregnancy, and/or key population status) was incomplete, the study is an intent-to-treat analysis using a “sharp” regression discontinuity design [18,19].

For each of the 6 countries in the analysis, we examined the unadjusted association between calendar date of enrollment in HIV care and rapid ART initiation. A discontinuity at the threshold of the date of national treat all policy adoption allowed for different slopes before and after the cutoff date. To estimate predicted outcomes and risk differences at the treat all threshold date, the following local linear regression models [21] were used:

$$E[Y_{i}|Z_{i}]=\beta_{0}+\beta_{1}*Z_{i}+\beta_{2}*1[Z_{i}\geq 0]+\beta_{3}*Z_{i}*1[Z_{i}\geq 0]$$

where *Y*_*i*_ is the patient-level outcome (rapid ART initiation), *Z*_*i*_ is the number of days between a patient’s enrollment date and the national treat all policy introduction date (negative if patient enrolled before the policy was introduced), and 1[*Z*_*i*_ ≥ 0] indicates whether a patient enrolled after the policy was introduced or not.

Data-driven Imbens–Kalyanaraman bandwidths were applied to minimize the mean squared error of the difference in predicted values at the threshold date of treat all introduction [22]. All observations within the bandwidth window were weighted equally (rectangular kernel). Sensitivity analyses were completed using 3 other bandwidth sizes (100, 200, and 300 days). In order to exclude possible threshold effects of prior eligibility expansions, only data for patients enrolling at least 90 days after the preceding ART eligibility expansion were included in bandwidth calculations and subsequent treat all regression discontinuity analysis. Prior to bandwidth calculations, patients enrolling in the 30 days immediately before treat all adoption were also excluded to ensure that there was no overlap in the outcome estimation windows (i.e., 30 days following enrollment) of those enrolling before and after the adoption of treat all. Consequently, to maintain continuity in *Z*_*i*_ (with a value of 0 corresponding to the threshold date of treat all introduction), 30 days were added to remaining enrollment dates preceding policy introduction. In effect, a patient who enrolled 31 days prior to policy introduction had *Z*_*i*_ = −1.

Pooled estimates of the risk difference at the treat all threshold were obtained from meta-analysis using a DerSimonian and Laird random-effects model, which computed a weighted average of all 6 countries’ effect estimates based on their standard errors [23].

#### Trends in rapid ART initiation before and after treat all adoption

To characterize trends in rapid ART initiation, slopes from linear regression models for the period before (starting 90 days after ART eligibility expansion to CD4 ≤ 500 cells/μl) and after the date of treat all adoption were compared and expressed as the percentage point (pp) change in rapid ART initiation per month.

#### Correlates of failure to initiate ART rapidly under treat all

To identify factors associated with failure to initiate ART rapidly among patients enrolling in HIV care under treat all, a multivariable, modified Poisson model with robust error variances was used [24]. A single model was fitted for all 6 countries, with age group, sex, and time between treat all adoption and enrollment (categorized into 0 to <3 months, 3 to <6 months, 6 to <12 months, and ≥12 months) as covariates. To represent other, unobserved confounders and health system features, the model also included a variable for country. Enrollment CD4 count was not included in the model, as it was only available for 37% of the patients.

Descriptive analyses were completed in SAS 9.3 and regression discontinuity analyses in Stata/IC version 14.2 [25].

The analysis plan was outlined in a May 2018 proposal to the IeDEA Executive Committee and is documented in S2 Text. The analysis of correlates of failure to initiate ART rapidly under treat all was not pre-specified.

## Results

### Sample characteristics

The 6 sub-Saharan African countries in the analysis adopted treat all policies between July and December 2016. A total of 814,603 patients met the study’s inclusion criteria, ranging from 11,176 in Burundi to 425,246 in Zambia. Overall, median age at enrollment across countries was 33 years. Over 60% of HIV care enrollees in all 6 countries were women, though the proportion decreased appreciably over time in Kenya and Rwanda (from 67.6% to 59.7% and from 64.8% to 56.7%, respectively, from period 1 to period 4) (Table 1).

**Table 1:** Baseline characteristics of patients enrolling in all 4 ART eligibility periods between 2004 and 2018 (*N =* 814,603).

Characteristic	Overall	Period 1 (CD4 ≤ 200/250 cells/μl)	Period 2 (CD4 ≤ 350 cells/μl)	Period 3 (CD4 ≤ 500 cells/μl)	Period 4 (treat all)
**Total *N***	814,603	389,416	216,832	169,822	38,533
**Burundi**					
Date of guideline expansion			October 2010	August 2014	September 2016
*N*	11,176	5,883	3,031	1,233	1,029
Median age (IQR) (years)	36 (28–44)	36 (29–45)	35 (28–44)	36 (27–45)	36 (27–45)
Percent female	63.8	65.7	61.6	63.3	59.7
Percent with enrollment CD4 count	19.1	13.3	33.6	16.0	13.4
Median enrollment CD4 count (IQR) (cells/μl)	302 (169–495)	257 (151–417)	323 (174–521)	347 (178–531)	432 (250–653)
**Kenya**					
Date of guideline expansion			November 2011	June 2014	July 2016
*N*	179,941	114,230	32,830	22,645	10,236
Median age (IQR) (years)	34 (27–41)	34 (28–42)	32 (26–40)	32 (26–41)	34 (28–43)
Percent female	66.5	67.6	66.0	64.6	59.7
Percent with enrollment CD4 count	73.5	81.4	69.7	57.3	32.6
Median enrollment CD4 count (IQR) (cells/μl)	242 (98–435)	228 (92–417)	267 (107–461)	302 (135–498)	272 (114–479)
**Malawi**					
Date of guideline expansion			July 2011	April 2014	July* 2016
*N*	84,558	37,215	24,803	18,881	3,659
Median age (IQR) (years)	32 (27–39)	33 (27–40)	32 (26–38)	32 (26–39)	32 (26–39)
Percent female	63.5	61.9	66.3	63.3	61.6
Percent with enrollment CD4 count	34.7	42.4	30.9	30.8	3.0
Median enrollment CD4 count (IQR) (cells/μl)	221 (115–352)	187 (93–295)	261 (146–391)	283 (156–431)	244 (154–411)
**Rwanda**					
Date of guideline expansion			August 2007	August 2011	July 2016
*N*	17,396	5,477	5,581	5,048	1,290
Median age (IQR) (years)	32 (26–39)	34 (28–40)	31 (26–38)	31 (25–38)	32 (26–39)
Percent female	61.5	64.8	59.3	61.7	56.7
Percent with enrollment CD4 count	75.3	67.2	79.8	79.9	72.2
Median enrollment CD4 count (IQR) (cells/μl)	350 (189–548)	278 (140–489)	364.5 (213–558)	384.5 (223–574)	391 (215–582)
**Uganda**					
Date of guideline expansion			June 2011	December 2013	November 2016
*N*	96,286	53,028	20,956	17,909	4,393
Median age (IQR) (years)	32 (26–39)	33 (27–40)	30 (25–38)	30 (24–37)	30 (24–37)
Percent female	62.8	64.2	61.7	60.5	61.3
Percent with enrollment CD4 count	72.5	69.7	79.3	78.7	49.3
Median enrollment CD4 count (IQR) (cells/μl)	278 (122–477)	236 (95–438)	315 (155–502)	337 (170–516)	334 (150–529)
**Zambia**					
Date of guideline expansion			June 2010	December 2013	December 2016
*N*	425,246	173,583	129,631	104,106	17,926
Median age (IQR) (years)	33 (28–40)	33 (28–40)	33 (27–40)	33 (27–40)	32 (26–39)
Percent female	62.0	62.9	61.6	61.3	60.8
Percent with enrollment CD4 count	67.2	79.8	66.7	52.4	34.0
Median enrollment CD4 count (IQR) (cells/μl)	226 (111–385)	195 (94–342)	253 (129–410)	270 (135–436)	289 (149–469)

*Adjusted based on documented policy rollout delays (see Methods).

Distribution of baseline characteristics among newly enrolling patients was similar just before and just after treat all adoption (excluding the 30 days immediately preceding the policy change) (S1 Table). No major discontinuity in the number of new enrollments around the date of treat all adoption was observed, consistent with our efforts to exclude sites with no pre-ART patient data (i.e., treat all did not result in an artificial increase in newly enrolling patients) (S1 Fig).

### Rapid ART initiation under treat all versus in previous ART eligibility periods (descriptive analysis)

Across the 6 countries, 81.6% of patients enrolling under treat all initiated treatment rapidly (within 30 days); 59.2% initiated on the day of enrollment in HIV care, and 67.1% within 7 days. Rapid ART initiation under treat all was highest in Malawi (88.9%) and Rwanda (86.9%), and lowest in Burundi (77.9%) (Table 2).

**Table 2:** Proportion of patients enrolling in HIV care under treat all and initiating ART on the day of enrollment, within 7 days, and within 30 days (“rapid ART initiation”).

Sample	Number of enrollees under treat all	Period of enrollment	Proportion of patients initiating ART		
			On the day of enrollment	Within 7 days of enrollment	Within 30 days of enrollment
**Overall**	38,533	May 2016–Jul 2018	59.2%	67.1%	81.6%
**By country**					
Burundi	1,029	Sep 2016–Jul 2018	32.7%	55.4%	77.9%
Kenya	10,236	Jul 2016–Jan 2018	63.6%	67.1%	83.1%
Malawi	3,659	Jul 2016–Nov 2016	69.5%	82.5%	88.9%
Rwanda	1,290	Jul 2016–Dec 2017	25.7%	56.7%	86.9%
Uganda	4,393	Nov 2016–Jan 2018	66.3%	71.7%	81.2%
Zambia	17,926	Dec 2016–Aug 2017	56.7%	64.2%	79.1%

Overall, rapid ART initiation increased by 25.9 pp under treat all, compared with the CD4 ≤ 500 cells/μl eligibility period (81.6% versus 55.7%). Larger increases in rapid ART initiation under treat all were observed among patients ≥25 years old than among those aged 16–24 years (26.5 versus 22.9 pp), whereas following the expansion to ART eligibility at CD4 ≤ 500 cells/μl, larger increases in rapid ART initiation were observed among those aged 16–24 years (23.9 versus 17.2 pp). No appreciable differences by sex were observed. The greatest increase in the proportion of patients initiating ART rapidly following expansion to treat all was observed in Rwanda (47.2 pp, from 39.7% to 86.9%) and Kenya (33.8 pp, from 49.3% to 83.1%) (S2 Fig).

**Fig 1:**
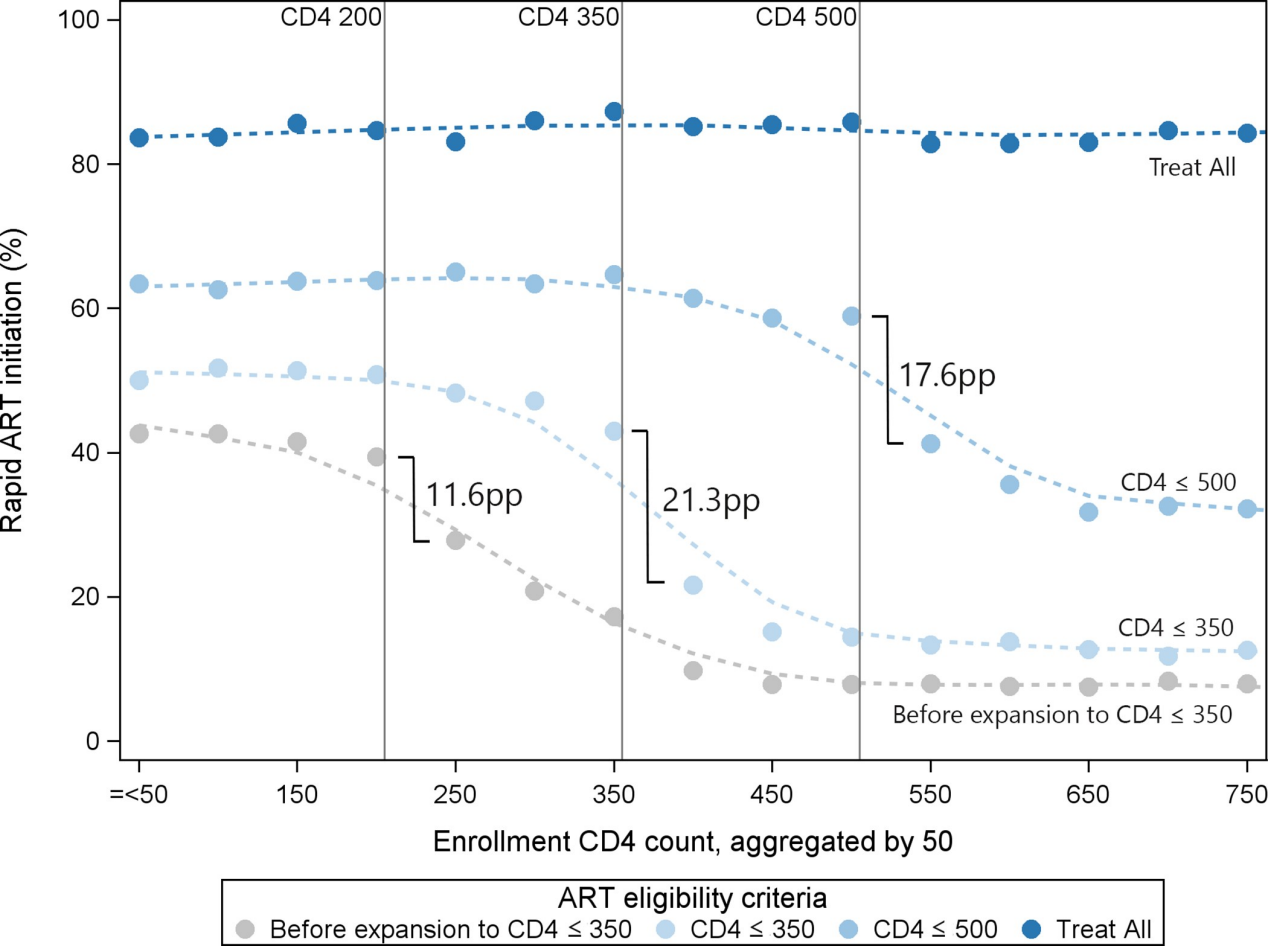
Rapid ART initiation (within 30 days of enrollment) across ART eligibility periods and enrollment CD4 counts (cells/μl).

Increases in rapid ART initiation were observed at every enrollment CD4 count level after each ART eligibility expansion. For earlier ART eligibility expansions, there were large differences in the proportion initiating ART among those with CD4 counts above the treatment eligibility threshold and those with CD4 counts below the threshold. For example, after eligibility expansion to CD4 ≤ 500 cells/μl, there was a 17.6-pp difference in rapid ART initiation between patients enrolling with CD4 counts of 451 to 500 cells/μl and those with CD4 counts of 501 to 550 cells/μl (58.9% versus 41.3%; medium blue points in Fig 1). In contrast, under treat all, there was little difference in rapid ART initiation across CD4 count levels, as indicated by the lack of any inflection point (dark blue points in Fig 1).

### Effect of treat all adoption on rapid ART initiation (regression discontinuity analysis)

**Fig 2:**
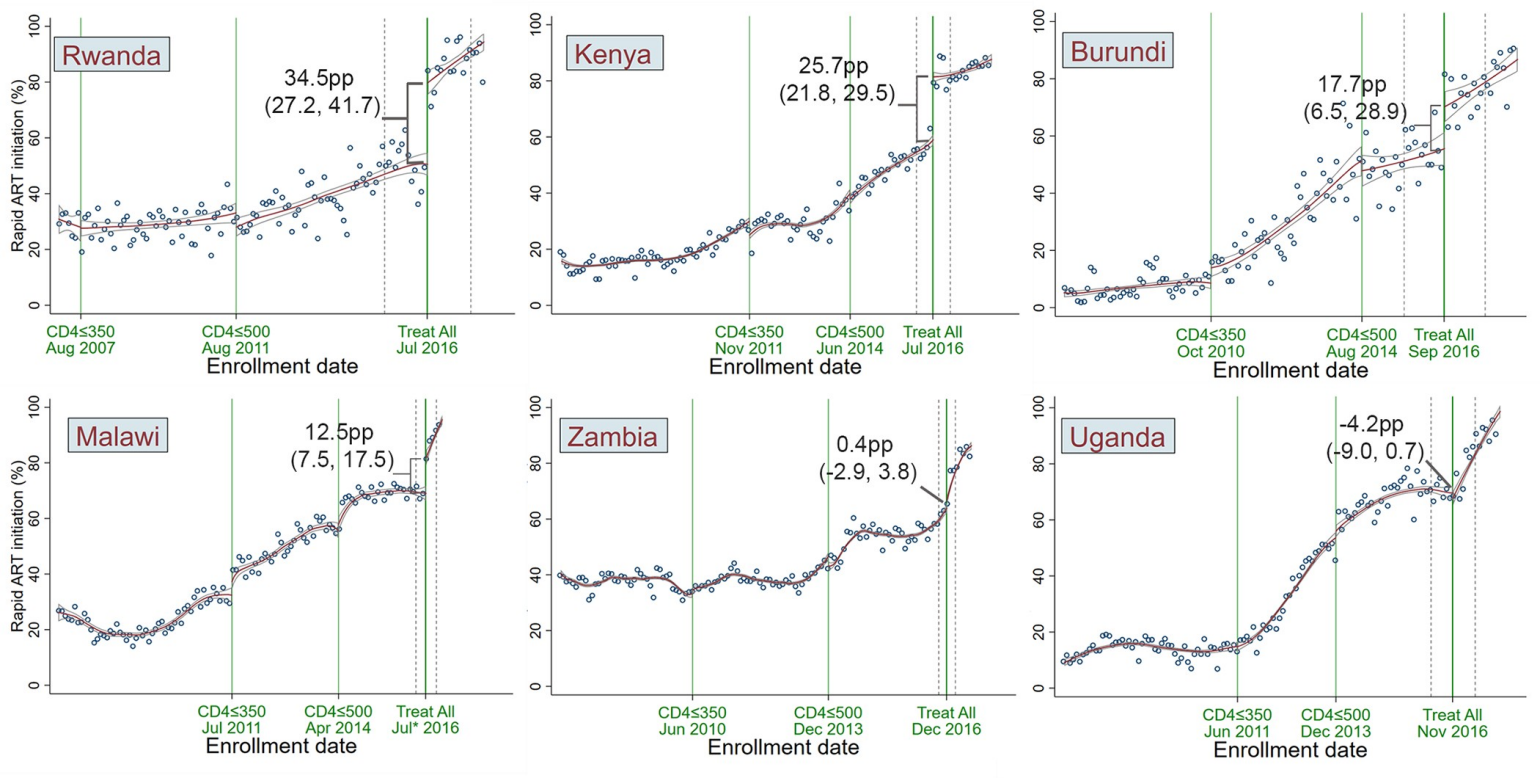
Rapid ART initiation (within 30 days of enrollment) by ART eligibility period and country, 2007–2018. Labels include effect sizes (percentage point [pp] change in the proportion of patients rapidly initiating ART) and 95% confidence intervals from the regression discontinuity analysis across the treat all adoption date threshold. Dotted lines on either side of the treat all date represent the width of the Imbens–Kalyanaraman bandwidth used in the regression discontinuity analysis. In order to comprehensively present observed trends, the graphs include the 30-day period preceding treat all adoption, which was excluded from regression discontinuity analysis (Table 3). The first 2 ART eligibility expansions (to CD4 ≤ 350 and ≤ 500 cells/μl) were not included in the regression discontinuity analysis, and data for the CD4 ≤ 350 cells/μl and CD4 ≤ 500 cells/μl eligibility periods are shown only for context. The plots include first degree local polynomial smooth curves intended for illustrative purposes and are distinct from the regression discontinuity models described in the Methods, from which effect estimates were derived. *Adjusted based on documented policy rollout delays (see Methods).

In 4 of the 6 countries, there was a statistically significant increase in rapid ART initiation immediately following national adoption of treat all. The effect was greatest in Rwanda, with a 34.5-pp increase (95% CI 27.2 to 41.7): 78.9% of patients enrolling immediately after treat all adoption initiated ART rapidly, compared with 44.4% of patients enrolling immediately before the policy change (a 77.7% relative increase). Increases were more moderate in Kenya (25.7 pp, 95% CI 21.8 to 29.5), Burundi (17.7 pp, 95% CI 6.5 to 28.9), and Malawi (12.5 pp, 95% CI 7.5 to 17.5). No statistically significant discontinuity effect was observed at the treat all threshold in Uganda (−4.2 pp, 95% CI −9.0 to 0.7) or Zambia (0.4 pp, 95% CI −2.9 to 3.8). The pooled estimate of effect at the treat all threshold was 14.2 pp (95% CI 2.2 to 26.2) (Table 3; Fig 2).

**Table 3:** Effect of enrollment under treat all on rapid ART initiation (within 30 days of enrollment), by country and pooled, and slopes before and after treat all adoption.

Measure	Pooled	Rwanda	Kenya	Burundi	Malawi	Zambia	Uganda
Risk difference at the treat all adoption threshold*	14.2	34.5	25.7	17.7	12.5	0.4	−4.2
95% CI	(2.2, 26.2)	(27.2, 41.7)	(21.8, 29.5)	(6.5, 28.9)	(7.5, 17.5)	(−2.9, 3.8)	(−9.0, 0.7)
*p*-Value	0.020	<0.001	<0.001	0.002	<0.001	0.804	0.090
Imbens–Kalyanaraman bandwidth, days		405	158	376	97	79	205
*N* within bandwidth		2,189	8,052	1,163	5,020	12,762	5,371
Predicted outcomes at the treat all threshold*							
Enrollment just before treat all adoption		44.4%	55.0%	55.7%	68.6%	62.4%	70.6%
Enrollment just after treat all adoption		78.9%	80.7%	73.4%	81.1%	62.8%	66.4%
Relative change after treat all adoption		77.7%	46.7%	31.8%	18.2%	0.6%	−5.9%
Slopes before and after treat all adoption**							
Percentage point change in rapid ART initiation per month before treat all adoption		0.4	0.6	0.6	0.1	0.2	0.4
Percentage point change in rapid ART initiation per month after treat all adoption		0.8	0.3	0.7	2.8	2.6	2.2
*p*-Value for difference of slopes		0.105	0.006	0.619	<0.001	<0.001	<0.001

*Risk difference and predicted outcomes at the treat all threshold are from regression discontinuity analysis. Effects are calculated at the modified guideline expansion threshold of 31 days before versus 1 day after treat all adoption.

**Slope comparison is from separate linear regression models comparing the period before treat all adoption (starting 90 days after ART eligibility expansion to CD4 ≤ 500 cells/μl) and after adoption.

Results of sensitivity analyses using other bandwidths were consistent with the findings based on the data-driven Imbens–Kalyanaraman bandwidth (S2 Table).

### Trends in rapid ART initiation before and after treat all adoption

After treat all adoption, the rate of change in rapid ART initiation increased most in Malawi (from 0.1 pp per month before treat all adoption to 2.8 pp per month afterwards), Zambia (from 0.2 pp to 2.6 pp), and Uganda (from 0.4 pp to 2.2 pp; all *p <* 0.001). A decrease in the rate was observed in Kenya (from 0.6 pp to 0.3 pp per month; *p =* 0.006).

### Correlates of failure to initiate ART rapidly under treat all

In post hoc analyses employing multivariable models restricted to patients enrolling under treat all, persons ages 16–24 years had an 18% greater risk of not starting ART rapidly, compared with those ≥25 years old (adjusted risk ratio [aRR] = 1.18, 95% CI 1.12 to 1.24), and men had a 12% higher risk than women (aRR = 1.12, 95% CI 1.07 to 1.17). There was an inverse relationship between time elapsed since treat all adoption and failure to initiate ART rapidly: At 12 months after treat all adoption, new enrollees had 47% lower risk of not starting ART rapidly compared to those who enrolled in the first 3 months after the policy was adopted (aRR = 0.53, 95% CI 0.48 to 0.58) (Table 4).

**Table 4:** Correlates of failure to initiate ART rapidly (within 30 days of enrollment) under treat all (*N =* 38,533).

Characteristic	*N*	RR (95% CI)	*p*-Value	aRR (95% CI)	*p*-Value
**Age**					
16–24 years	7,259	1.15 (1.10, 1.21)	<0.001	1.18 (1.12–1.24)	<0.001
≥25 years	31,274	Ref		Ref	
**Sex**					
Male	15,220	1.08 (1.04, 1.13)	<0.001	1.12 (1.07–1.17)	<0.001
Female	23,313	Ref		Ref	
**Time between treat all adoption and enrollment**					
0 to <3 months	14,075	Ref	<0.001	Ref	<0.001
3 to <6 months	12,035	0.74 (0.70, 0.77)		0.71 (0.68–0.75)	
6 to <12 months	8,737	0.67 (0.63, 0.71)		0.63 (0.59–0.66)	
≥12 months	3,686	0.57 (0.53, 0.63)		0.53 (0.48–0.58)	

Model also adjusted for country.

aRR, adjusted risk ratio; RR, risk ratio.

## Discussion

We found that adoption of treat all policies at the national level in 6 African countries was followed by appreciable increases in the proportion of patients initiating ART rapidly (within 30 days of enrollment) in diverse, largely public-sector service delivery settings. Regression discontinuity analysis found marked increases in rapid ART initiation in most of the countries immediately after the national adoption of treat all. Our study provides evidence, outside of a controlled research environment, of a causal effect of national adoption of the treat all policy on sustained improvements in rapid ART initiation following HIV care enrollment, with no apparent negative effects on ART initiation for those eligible under prior treatment guidelines.

In half of the countries in the analysis, immediate relative increases in rapid ART initiation at the regression discontinuity threshold (ranging from 31.8% to 77.7% in Burundi, Kenya, and Rwanda) were larger than the 26.7% increase in the number of ART initiators predicted for a 1-year period after treat all adoption by a recent empirical modeling study for South Africa [26], and similar to those observed in a recent trial in Eswatini [12] and a recent analysis of ART uptake in Rwanda following national adoption of treat all [27].

Improvements were sustained or further amplified following national adoption of treat all. In Malawi, Uganda, and Zambia, the rate of change in rapid ART initiation increased sharply in the months following the date of treat all adoption, suggesting that implementation of expanded treatment eligibility guidelines may have been phased in or delayed. This was particularly true in Uganda and Zambia, where no effect was observed at the threshold. Lack of an immediate effect may also suggest that there were few newly eligible patients at the time when treat all was adopted, or it may reflect regression to the mean, given already-high levels of rapid ART initiation in the months leading up to eligibility expansion.

Previous studies have reported lower rates of ART initiation among men [9,28] and young adults [6,29], compared to women and older adults. Similarly, while rapid ART initiation rates improved for both groups following the adoption of treat all, our analysis found that men and young adults remained at greater risk of not initiating ART rapidly. While we found that the risk of failing to rapidly initiate ART decreased markedly with time following national adoption of treat all, as more follow-up data become available, future research should assess whether age and sex disparities in ART initiation diminish over time.

Overall, there were no appreciable differences in rates of rapid ART initiation across different enrollment CD4 counts. If observed, such differences would have suggested that the sickest patients were being “crowded out” by patients with less advanced disease under treat all. While these findings are consistent with research assessing the impacts of prior ART eligibility expansions [6,7,30], there may be variations at the country or site level that we were unable to assess in stratified analyses, largely because substantially fewer patients under treat all have enrollment CD4 counts measured before ART initiation.

A major strength of our study is the use of a regression discontinuity design and service delivery data that reflect diverse real-world implementation settings in sub-Saharan Africa, which provide support for a causal interpretation of the association between expanded ART eligibility under treat all and increases in rapid ART uptake [20]. The use of data-driven Imbens–Kalyanaraman optimal bandwidths and sensitivity analyses with 3 other constant bandwidths enabled us to generate robust effect estimates with minimal risk of researcher bias [22].

However, a limitation of our study is incomplete data on potential ART eligibility criteria, such as clinical status (including TB coinfection) and pregnancy, as well as enrollment CD4 count. Such data would have better elucidated the actual ART eligibility status of patients enrolling before treat all. These gaps precluded the implementation of a “fuzzy” regression discontinuity design, which would better reflect the probabilistic distribution of ART eligibility in the pre-treat-all sample [18]. Similarly, we were unable to assess rapid ART initiation specifically among patients previously eligible for ART, which could have provided insights into barriers to rapid ART initiation in the pre-treat-all era (e.g., capacity constraints and/or delays in eligibility ascertainment versus adherence to policy at the time). In addition, the limited availability of data on patient characteristics other than age, sex, and enrollment CD4 count restricted our ability to assess whether patients on each side of the regression discontinuity threshold were similar with respect to other pre-treatment covariates.

Another limitation is that the exact date of treat all policy adoption at the site level is unknown for many IeDEA sites, and delays in site-level implementation of expanded treatment eligibility guidelines likely varied within and across countries. Such potential non-differential exposure misclassification would be expected to attenuate observed effects and may have contributed to the observed results in Uganda and Zambia (i.e., no increase in rapid ART initiation at the regression discontinuity threshold, but increases in ART initiation rates with increasing time after national adoption of treat all). At the same time, if facilities contributing data to an international collaboration such as IeDEA have a greater capacity to rapidly implement policy changes, the threshold effects we found may overestimate the effects of the adoption of treat all in the respective countries.

These findings are nonetheless important, given previous research showing that loss to care between diagnosis and ART initiation is the most common “breakpoint” in the HIV care continuum [31], as well as recent evidence of improved retention in care among those who are immediately eligible for ART [4,11,19]. As more data on HIV care under treat all become available, research should assess longer-term patient outcomes, including rate of and time to viral load suppression among patients who immediately initiate ART. Recent findings from a trial in Eswatini have reported large improvements in viral load suppression under treat all [11]; however, additional analyses utilizing real-world service delivery data from diverse country contexts and quasi-experimental designs will be important for deriving generalizable effect estimates of the individual- and population-level benefits of treat all policies. Country-specific analyses incorporating multiple change points for treatment eligibility expansions could provide additional insight into the relative impacts of distinct policies over time in diverse contexts. Further research into health system constraints, demand-side barriers, and underserved populations is also critical for understanding the drivers of between-country differences in the effect of treat all policies on rapid ART initiation. Equally important is implementation research to identify effective strategies for increasing the uptake of HIV testing and linkage to care [26,32], as well as for optimizing patient outcomes along the HIV care cascade under treat all [33]. Finally, as more follow-up data under treat all become available, questions around retention and viral suppression, as well as treatment failure and subsequent regimen switches, will be important to examine.

In conclusion, our study demonstrates a strong and sustained effect of national-level adoption of treat all policies on rapid ART initiation in diverse service delivery settings across 6 sub-Saharan African countries. This provides further evidence of treat all being a key strategy towards the achievement of UNAIDS 90-90-90 targets.

## Supporting information

S1 STROBE checklist (DOCX)

S1 Fig Distribution of HIV care enrollment in the year before and after treat all adoption. (TIF)

S2 Fig Rapid ART initiation (within 30 days of enrollment) across enrollment/patient characteristics. (A) ART eligibility period, (B) age group, (C) sex, and (D) country.
(TIF)

S1 Table Covariate balance at the treat all adoption threshold. (DOCX)

S2 Table Sensitivity analyses with other bandwidth sizes. (DOCX)

S1 Text Full membership of the IeDEA consortium. (DOCX)

S2 Text Analysis concept sheet. (DOCX)

## Abbreviations

aRR: adjusted risk ratio
ART: antiretroviral therapy
IeDEA: International epidemiology Databases to Evaluate AIDS
PLWH: people living with HIV
pp: percentage point
UNAIDS: Joint United Nations Programme on HIV/AIDS
WHO: World Health Organization
