# Supplementary figures and images for: Changes in rapid HIV treatment initiation after national “treat all” policy adoption in 6 sub-Saharan African countries: Regression discontinuity analysis
Source: PLoS Med. 2019 Jun 10;16(6):e1002822. doi: 10.1371/journal.pmed.1002822 (PMC6557472; doi:10.1371/journal.pmed.1002822)

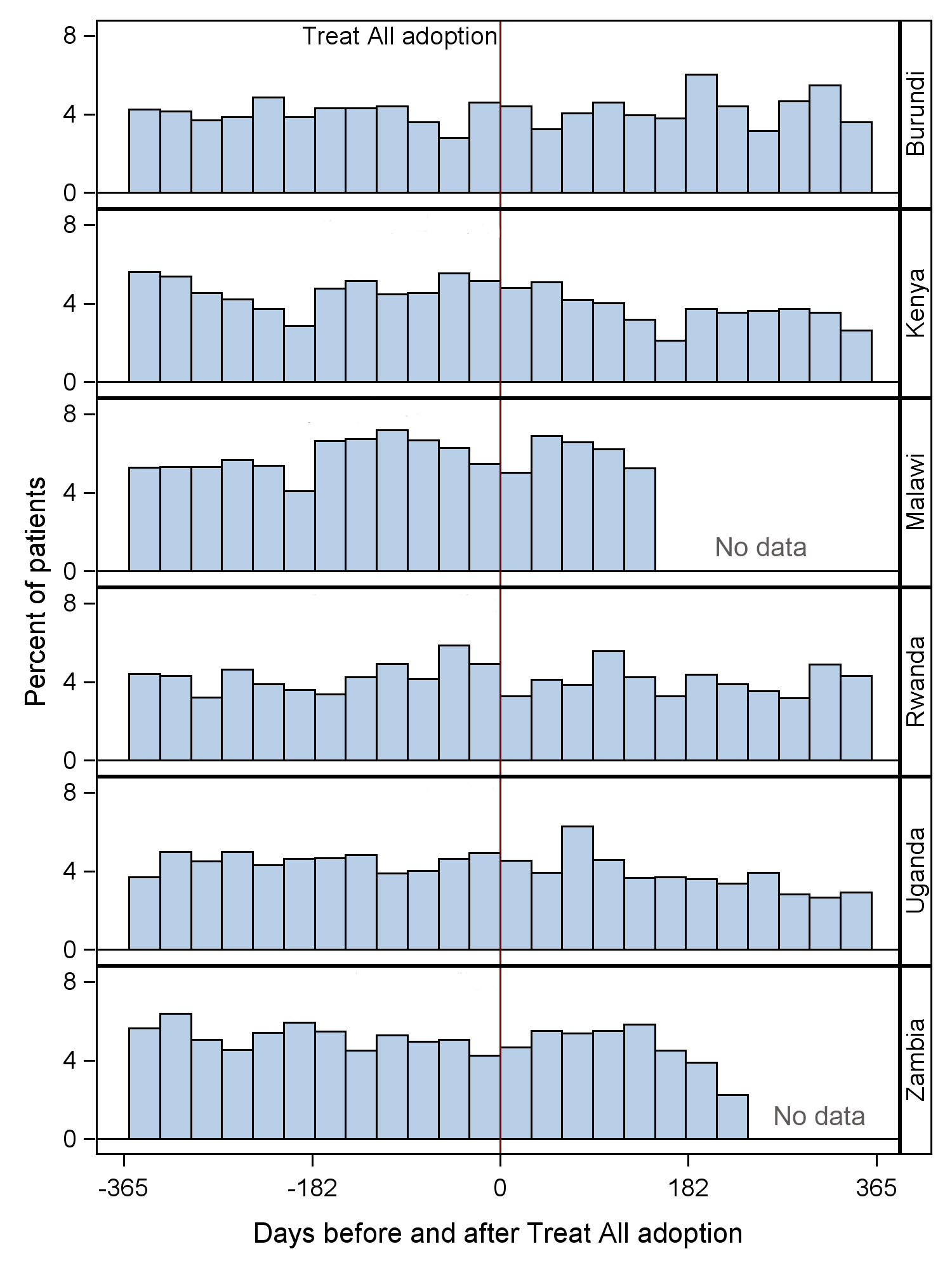

Supplement: S1 Fig — (TIF) [file pmed.1002822.s002.tif]

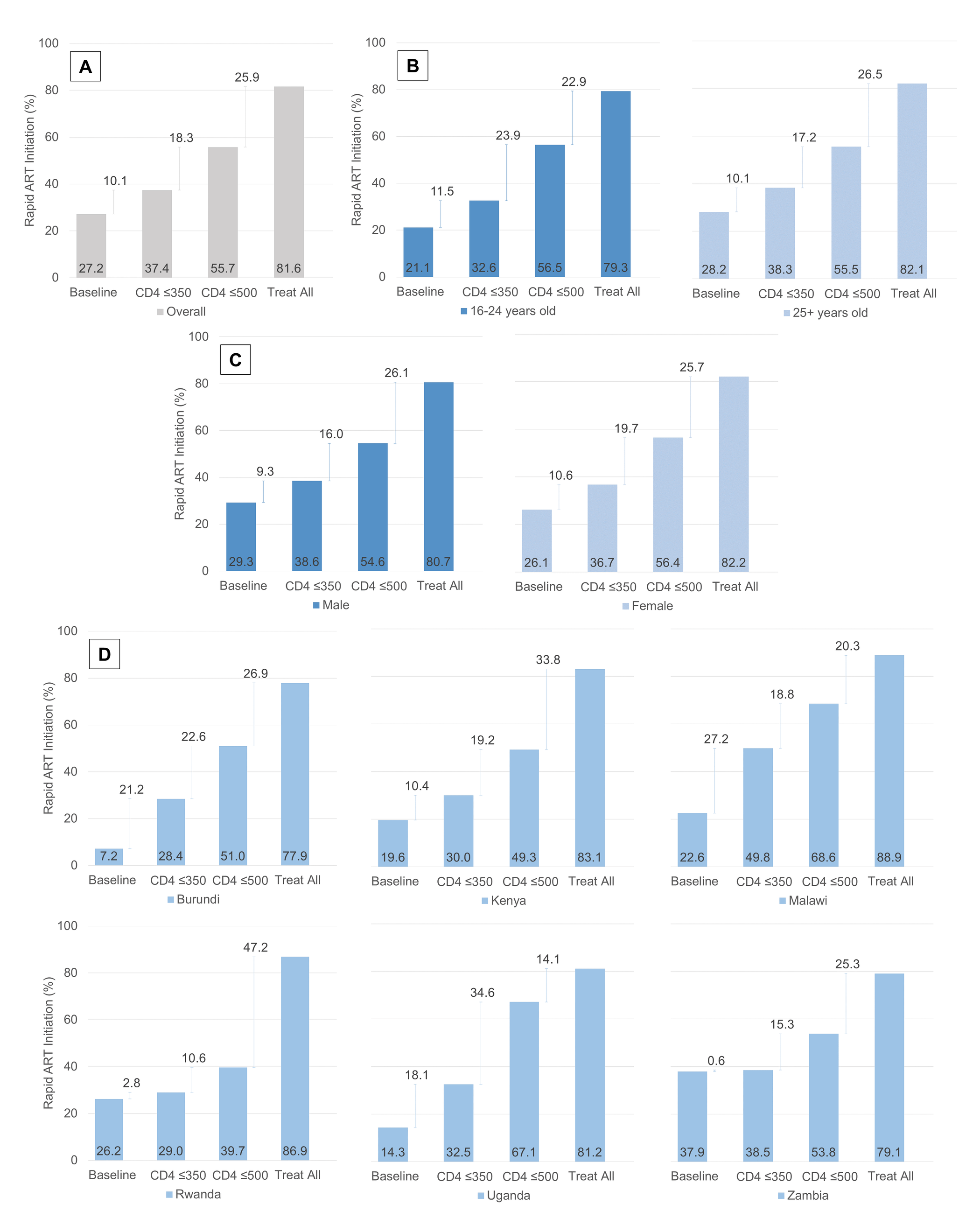

Supplement: S2 Fig — (A) ART eligibility period, (B) age group, (C) sex, and (D) country. (TIF) [file pmed.1002822.s003.tif]
