# Supplementary material for: Changes in rapid HIV treatment initiation after national “treat all” policy adoption in 6 sub-Saharan African countries: Regression discontinuity analysis
Source: PLoS Med. 2019 Jun 10;16(6):e1002822. doi: 10.1371/journal.pmed.1002822 (PMC6557472; doi:10.1371/journal.pmed.1002822)
Supplement: S1 Table — (DOCX) [file pmed.1002822.s004.docx]

|  | **Predicted means for**  **patients enrolling:** | | **Difference at the threshold (95% CI)** | **p-value** | **IK* bandwidth (days)** | **N** |
| --- | --- | --- | --- | --- | --- | --- |
|  | **Just before Treat All adoption** | **Just after Treat All adoption** |  |  |  |  |
| Burundi |  |  |  |  |  |  |
| Age | 38.6 | 36.8 | -1.8 (-4.9, 1.3) | 0.248 | 353 | 1,087 |
| Female | 63.3% | 65.4% | 2.1 (-9.0, 13.2) | 0.710 | 407 | 1,240 |
| Has enrolment CD4 count** | 39.0% | 26.8% | -12.1 (-27.0, 2.7) | 0.110 | 203 | 626 |
| Enrolment CD4 count | 457.5 | 447.5 | -10.0 (-203.6, 183.7) | 0.910 | 219 | 158 |
| Kenya |  |  |  |  |  |  |
| Age | 34.6 | 34.9 | 0.3 (-0.6, 1.2) | 0.534 | 181 | 8,674 |
| Female | 61.8% | 61.3% | -0.5 (-4.0, 3.0) | 0.784 | 217 | 10,140 |
| Has enrolment CD4 count** | 62.6% | 50.1% | -12.5 (-17.2, -7.8) | <0.001 | 117 | 6,232 |
| Enrolment CD4 count | 358.4 | 382.5 | 24,1 (-11.7, 59.9) | 0.187 | 137 | 3,793 |
| Malawi |  |  |  |  |  |  |
| Age | 33.4 | 33.9 | 0.6 (-0.3, 1.5) | 0.214 | 178 | 8,163 |
| Female | 63.2% | 66.1% | 2.9 (-3.0, 8.8) | 0.336 | 94 | 4,674 |
| Has enrolment CD4 count** | 17.2% | 9.4% | -7.8 (-11.4, -4.9) | <0.001 | 97 | 5,079 |
| Enrolment CD4 count | 265.6 | 312.5 | 46.9 (-41.1, 135.0) | 0.296 | 75 | 443 |
| Rwanda |  |  |  |  |  |  |
| Age | 33.7% | 32.3% | -1.4 (-3.1, 0.3) | 0.115 | 368 | 1,964 |
| Female | 62.3% | 60.8% | -1.5 (-10.3, 7.1) | 0.726 | 356 | 1,891 |
| Has enrolment CD4 count** | 82.3% | 74.4% | -7.9 (-14.1, -1.7) | 0.013 | 503 | 2,666 |
| Enrolment CD4 count | 432.9 | 446.8 | 13.8 (-40.0, 67.7) | 0.614 | 384 | 1,624 |
| Uganda |  |  |  |  |  |  |
| Age | 31.2 | 30.7 | -0.5 (-1.5, 0.5) | 0.340 | 241 | 6,236 |
| Female | 63.2% | 63.9% | 0.7 (-3.7, 5.1) | 0.767 | 283 | 7,343 |
| Has enrolment CD4 count** | 63.4% | 42.1% | -21.3 (-27.2, -15.4) | <0.001 | 160 | 4,293 |
| Enrolment CD4 count | 409.9 | 398.9 | -11.0 (-53.0, 31.0) | 0.609 | 214 | 3,530 |
| Zambia |  |  |  |  |  |  |
| Age*** | 34.2 | 33.6 | -0.6 (-1.0, -0.2) | 0.002 | 338 | 46,387 |
| Female | 59.9% | 60.8% | 0.9 (-1.3, 3.2) | 0.414 | 180 | 29,855 |
| Has enrolment CD4 count | 46.3% | 44.5% | -1.8 (-5.0, 1.5) | 0.282 | 90 | 14,689 |
| Enrolment CD4 count | 319.9 | 300.3 | -19.6 (-40.7, 1.5) | 0.068 | 121 | 8,224 |
| * Imbens-Kalyanaraman bandwidth | | | | | | |
| ** Decreases in CD4 count testing after UTT adoption are expected and a direct consequence of a Treat All policy | | | | | | |
| *** Statistical significance of change in mean age at the Treat All threshold in Zambia is likely driven by the large sample size, and the low magnitude of effect suggests that it is not meaningful | | | | | | |
