# Supplementary material for: Changes in rapid HIV treatment initiation after national “treat all” policy adoption in 6 sub-Saharan African countries: Regression discontinuity analysis
Source: PLoS Med. 2019 Jun 10;16(6):e1002822. doi: 10.1371/journal.pmed.1002822 (PMC6557472; doi:10.1371/journal.pmed.1002822)
Supplement: S2 Table — (DOCX) [file pmed.1002822.s005.docx]

| **Bandwidth = 100 days** | **Pooled** | **Rwanda** | **Kenya** | **Burundi** | **Malawi** | **Zambia** | **Uganda** |
| --- | --- | --- | --- | --- | --- | --- | --- |
| Risk difference at the Treat All adoption threshold | 14.9 | 42.9 | 19.0 | 13.1 | 12.5 | 4.0 | 7.0 |
| 95% CI | (6.8, 23.0) | (28.4, 57.4) | (14.4, 23.7) | (-10.8, 37.0) | (7.7, 17.4) | (1.0, 7.0) | (-0.1, 14.0) |
| p-value |  | <0.001 | <0.001 | 0.282 | <0.001 | 0.008 | 0.053 |
| N within bandwidth |  | 574 | 5,335 | 277 | 5,207 | 16,392 | 2,710 |
| **Bandwidth = 200 days** | **Pooled** | **Rwanda** | **Kenya** | **Burundi** |  | **Zambia** | **Uganda** |
| Risk difference at the Treat All adoption threshold | 15.8 | 39.0 | 24.6 | 12.9 |  | 8.3 | -3.7 |
| 95% CI | (4.2, 27.4) | (28.5, 49.5) | (21.2, 28.1) | (-2.9, 28.8) |  | (6.3, 10.4) | (-8.6, 1.2) |
| p-value |  | <0.001 | <0.001 | 0.110 |  | <0.001 | 0.139 |
| N within bandwidth |  | 1,058 | 9,412 | 604 |  | 33,067 | 5,244 |
| **Bandwidth = 300 days^*^** | **Pooled** | **Rwanda** | **Kenya** | **Burundi** |  | **Zambia** | **Uganda** |
| Risk difference at the Treat All adoption threshold | 19.3 | 36.3 | 25.5 | 19.2 |  |  | -3.0 |
| 95% CI | (0.8, 37.9) | (27.7, 44.9) | (22.7, 28.2) | (6.4, 32.0) |  |  | (-8.6, 1.2) |
| p-value |  | <0.001 | <0.001 | 0.003 |  |  | 0.132 |
| N within bandwidth |  | 1,564 | 14,078 | 910 |  |  | 7,674 |
| * Bandwidth of size 300 days not used in Malawi and Zambia, as well as 200 days in Malawi, due to short follow-up time available in those countries (enrollments eligible for inclusion in the analysis terminate at 152 and 229 days after Treat All adoption, respectively). Pooled estimates for these bandwidths are not comparable with the pooled 100 day bandwidth estimate, because not all six countries were included in them. | | | | | | | |
