# Supplementary material for: Changes in rapid HIV treatment initiation after national “treat all” policy adoption in 6 sub-Saharan African countries: Regression discontinuity analysis
Source: PLoS Med. 2019 Jun 10;16(6):e1002822. doi: 10.1371/journal.pmed.1002822 (PMC6557472; doi:10.1371/journal.pmed.1002822)
Supplement: S1 Text — (DOCX) [file pmed.1002822.s006.docx]

**Asia-Pacific**

**TAHOD (Adult Asia)**

**Funding acknowledgement**

The TREAT Asia HIV Observational Database is an initiative of TREAT Asia, a program of amfAR, The Foundation for AIDS Research, with support from the U.S. National Institutes of Health’s National Institute of Allergy and Infectious Diseases, the Eunice Kennedy Shriver National Institute of Child Health and Human Development, the National Cancer Institute, the National Institute of Mental Health, and the National Institute on Drug Abuse, as part of the International Epidemiology Databases to Evaluate AIDS (IeDEA; U01AI069907). The Kirby Institute is funded by the Australian Government Department of Health and Ageing, and is affiliated with the Faculty of Medicine, UNSW Sydney. The content of this publication is solely the responsibility of the authors and does not necessarily represent the official views of any of the governments or institutions mentioned above.

**Site investigators and study team:**

PS Ly* and V Khol, National Center for HIV/AIDS, Dermatology & STDs, Phnom Penh, Cambodia; FJ Zhang*, HX Zhao and N Han, Beijing Ditan Hospital, Capital Medical University, Beijing, China; MP Lee*, PCK Li, W Lam and YT Chan, Queen Elizabeth Hospital, Hong Kong SAR, China; N Kumarasamy*‡, S Saghayam and C Ezhilarasi, Chennai Antiviral Research and Treatment Clinical Research Site (CART CRS), YRGCARE Medical Centre, VHS, Chennai, India; S Pujari*, K Joshi , S Gaikwad and A Chitalikar, Institute of Infectious Diseases, Pune, India; TP Merati*, DN Wirawan and F Yuliana, Faculty of Medicine Udayana University & Sanglah Hospital, Bali, Indonesia; E Yunihastuti*, D Imran and A Widhani, Faculty of Medicine Universitas Indonesia - Dr. Cipto Mangunkusumo General Hospital, Jakarta, Indonesia; J Tanuma*, S Oka and T Nishijima, National Center for Global Health and Medicine, Tokyo, Japan; JY Choi*, Na S and JM Kim, Division of Infectious Diseases, Department of Internal Medicine, Yonsei University College of Medicine, Seoul, South Korea; BLH Sim*, YM Gani, and NB Rudi, Hospital Sungai Buloh, Sungai Buloh, Malaysia; A Kamarulzaman*, SF Syed Omar, S Ponnampalavanar and I Azwa, University Malaya Medical Centre, Kuala Lumpur, Malaysia; R Ditangco*, MK Pasayan and ML Mationg, Research Institute for Tropical Medicine, Muntinlupa City, Philippines; YJ Chan*, WW Ku and PC Wu, Taipei Veterans General Hospital, Taipei, Taiwan; OT Ng*†, PL Lim, LS Lee and Z Ferdous, Tan Tock Seng Hospital, Singapore; A Avihingsanon*, S Gatechompol, P Phanuphak and C Phadungphon, HIV-NAT/Thai Red Cross AIDS Research Centre, Bangkok, Thailand; S Kiertiburanakul*, A Phuphuakrat, L Chumla and N Sanmeema, Faculty of Medicine Ramathibodi Hospital, Mahidol University, Bangkok, Thailand; R Chaiwarith*, T Sirisanthana, W Kotarathititum and J Praparattanapan, Research Institute for Health Sciences, Chiang Mai, Thailand; S Khuwuwan*, P Kantipong and P Kambua, Chiangrai Prachanukroh Hospital, Chiang Rai, Thailand; KV Nguyen*, HV Bui, DTH Nguyen and DT Nguyen, National Hospital for Tropical Diseases, Hanoi, Vietnam; CD Do*, AV Ngo and LT Nguyen, Bach Mai Hospital, Hanoi, Vietnam; AH Sohn*, JL Ross* and B Petersen, TREAT Asia, amfAR - The Foundation for AIDS Research, Bangkok, Thailand; MG Law*, A Jiamsakul*, R Bijker and D Rupasinghe, The Kirby Institute, UNSW Sydney, NSW, Australia.

* TAHOD Steering Committee member; † Steering Committee Chair; ‡ co-Chair.

**Combined Asia and Australia – Adults**

The TREAT Asia HIV Observational Database and the Australian HIV Observational Database are initiatives of TREAT Asia, a program of amfAR, The Foundation for AIDS Research, with support from the U.S. National Institutes of Health’s National Institute of Allergy and Infectious Diseases, the Eunice Kennedy Shriver National Institute of Child Health and Human Development, the National Cancer Institute, the National Institute of Mental Health, and the National Institute on Drug Abuse, as part of the International Epidemiology Databases to Evaluate AIDS (IeDEA; U01AI069907). The Kirby Institute is funded by the Australian Government Department of Health and Ageing, and is affiliated with the Faculty of Medicine, UNSW Sydney. The content of this publication is solely the responsibility of the authors and does not necessarily represent the official views of any of the governments or institutions mentioned above.

**The TREAT Asia Pediatric HIV Network**

The TREAT Asia Pediatric HIV Observational Database is an initiative of TREAT Asia, a program of amfAR, The Foundation for AIDS Research, with support from the US National Institutes of Health’s National Institute of Allergy and Infectious Diseases, the Eunice Kennedy Shriver National Institute of Child Health and Human Development, National Cancer Institute, National Institute of Mental Health, and National Institute on Drug Abuse as part of the International Epidemiology Databases to Evaluate AIDS (IeDEA; U01AI069907). The Kirby Institute is funded by the Australian Government Department of Health and Ageing, and is affiliated with the Faculty of Medicine, UNSW Australia. The content of this publication is solely the responsibility of the authors and does not necessarily represent the official views of any of the governments or institutions mentioned above.

**Site investigators and cohorts:**

PS Ly*, and V Khol, National Centre for HIV/AIDS, Dermatology and STDs, Phnom Penh, Cambodia; J Tucker, New Hope for Cambodian Children, Phnom Penh, Cambodia; N Kumarasamy*, and E Chandrasekaran, YRGCARE Medical Centre, CART CRS, Chennai, India; A Kinikar*, V Mave, S Nimkar and I Marbaniang, BJ Medical College and Sassoon General Hospitals, Maharashtra, India; DK Wati*, D Vedaswari, and IB Ramajaya, Sanglah Hospital, Udayana University, Bali, Indonesia; N Kurniati*, and D Muktiarti, Cipto Mangunkusumo – Faculty of Medicine Universitas Indonesia, Jakarta, Indonesia; SM Fong*, M Lim, and F Daut, Hospital Likas, Kota Kinabalu, Malaysia; NK Nik Yusoff*, and P Mohamad, Hospital Raja Perempuan Zainab II, Kelantan, Malaysia; TJ Mohamed*‡, and MR Drawis, Pediatric Institute, Hospital Kuala Lumpur, Kuala Lumpur, Malaysia; R Nallusamy*, and KC Chan, Penang Hospital, Penang, Malaysia; T Sudjaritruk*, V Sirisanthana, and L Aurpibul, Department of Pediatrics, Faculty of Medicine, and Research Institute for Health Sciences, Chiang Mai University, Chiang Mai, Thailand; R Hansudewechakul*, P Ounchanum, S Denjanta, and A Kongphonoi, Chiangrai Prachanukroh Hospital, Chiang Rai, Thailand; P Lumbiganon*, P Kosalaraksa, P Tharnprisan, and T Udomphanit, Division of Infectious Diseases, Department of Pediatrics, Faculty of Medicine, Khon Kaen University, Khon Kaen, Thailand; G Jourdain, PHPT-IRD UMI 174 (Institut de recherche pour le développement and Chiang Mai University), Chiang Mai, Thailand; T Puthanakit*, S Anugulruengkit, W Jantarabenjakul and R Nadsasarn, Department of Pediatrics, Faculty of Medicine and Research Unit in Pediatric and Infectious Diseases, Chulalongkorn University, Bangkok, Thailand; K Chokephaibulkit*†, K Lapphra, W Phongsamart, and S Sricharoenchai, Department of Pediatrics, Faculty of Medicine Siriraj Hospital, Mahidol University, Bangkok, Thailand; KH Truong*, QT Du, and CH Nguyen, Children’s Hospital 1, Ho Chi Minh City, Vietnam; VC Do*, TM Ha, and VT An Children’s Hospital 2, Ho Chi Minh City, Vietnam; LV Nguyen*, DTK Khu, AN Pham, and LT Nguyen, National Hospital of Pediatrics, Hanoi, Vietnam; ON Le, Worldwide Orphans Foundation, Ho Chi Minh City, Vietnam; AH Sohn*, JL Ross, and T Suwanlerk, TREAT Asia/amfAR -- The Foundation for AIDS Research, Bangkok, Thailand; MG Law*, and A Kariminia, The Kirby Institute, UNSW Australia, Sydney, Australia; (*Steering Committee members; † Current Steering Committee Chair; ‡ co-Chair).

**CCASAnet**

**Procedure for CCASAnet Citation**

*v. 30 October 2018*

In all publications, please cite as appropriate the **Caribbean, Central and South America network for HIV epidemiology (CCASAnet)**. CCASAnet contributes to our research environment in many ways, including intellectual exchange, mentoring, consultation, data, collaboration, etc., that extend far beyond direct funding for individual projects. We suggest the following wording:

"This work was supported by the NIH-funded Caribbean, Central and South America network for HIV epidemiology (CCASAnet), a member cohort of the International Epidemiologic Databases to Evaluate AIDS (leDEA) (U01AI069923). This award is funded by the following institutes: Eunice Kennedy Shriver National Institute Of Child Health & Human Development (NICHD), National Cancer Institute (NCI), National Institute Of Allergy And Infectious Diseases (NIAID), National Institute Of Mental Health (NIMH), and the Office Of The Director, National Institutes Of Health (OD).” ***See note below on additional funding citations, if relevant.*

**For projects that use **RePORT-Brazil tuberculosis data**, please also include:

R01AI120790, “Predictors of Treatment Toxicity, Failure, and Relapse in HIV-Related Tuberculosis”

**For projects that use **data or methods from Bryan Shepherd’s R01**, please also include:

R01AI131771, “Statistical Methods for Correlated Outcome and Covariate Errors in Studies of HIV/AIDS”

**For projects involving **Peter Rebeiro**, please also include:

K01AI131895, “The HIV Care Continuum and Health Policy: Changes Through Context and Geography”

**For projects involving **Jessica Castilho**, please also include:

K23AI120875, “The Dynamics of HIV, Aging, and Lymphocyte Exhaustion”

**For projects utilizing **resources from the IeDEA Harmonist Toolkit**, please also include:

R24AI124872, “Harmonist: A Scalable Toolkit for Standardizing and Coordinating Data Sharing Across International Research Networks”

If you wish to add the citation to grant U01AI069923 to a publication that has already been published, you may need to contact the National Library of Medicine, at [**nlmcustservmbx@nlm.nih.gov**](mailto:nlmcustservmbx@nlm.nih.gov) and ask them to connect grant number U01AI069923 to your particular PMID or PMCID (just linking grant to manuscript in "My NCBI” doesn’t seem to work).

Acknowledgement List

**IeDEA Caribbean, Central, and South America (CCASAnet):**

**Fundación Huésped, Argentina:** Pedro Cahn, Carina Cesar, Valeria Fink, Omar Sued, Emanuel Dell’Isola, Hector Perez, Jose Valiente, Cleyton Yamamoto.

**Instituto Nacional de Infectologia-Fiocruz, Brazil:** Beatriz Grinsztejn, Valdilea Veloso, Paula Luz, Raquel de Boni, Sandra Cardoso Wagner, Ruth Friedman, Ronaldo Moreira.

**Universidade Federal de Minas Gerais, Brazil:** Jorge Pinto, Flavia Ferreira, Marcelle Maia.

**Universidade Federal de São Paulo, Brazil:** Regina Célia de Menezes Succi, Daisy Maria Machado, Aida de Fátima Barbosa Gouvêa.

**Fundación Arriarán, Chile**: Marcelo Wolff, Claudia Cortes, Maria Fernanda Rodriguez, Gladys Allendes.

**Les Centres GHESKIO, Haiti:** Jean William Pape, Vanessa Rouzier, Adias Marcelin, Christian Perodin.

**Hospital Escuela Universitario, Honduras**: Marco Tulio Luque.

**Instituto Hondureño de Seguridad Social, Honduras:** Denis Padgett.

**Instituto Nacional de Ciencias Médicas y Nutrición Salvador Zubirán, Mexico**: Juan Sierra Madero, Brenda Crabtree Ramirez, Paco Belaunzaran, Yanink Caro Vega.

**Instituto de Medicina Tropical Alexander von Humboldt, Peru**: Eduardo Gotuzzo, Fernando Mejia, Gabriela Carriquiry.

**Vanderbilt University Medical Center, USA:** Catherine C McGowan, Bryan E Shepherd, Timothy Sterling, Karu Jayathilake, Anna K Person, Peter F Rebeiro, Jessica Castilho, Stephany N Duda, Fernanda Maruri, Hilary Vansell, Cathy Jenkins, Ahra Kim, Sarah Lotspeich.

**Central Africa (CA-IeDEA)**

**Funding acknowledgement**

Each publication, press release, or other document about research supported by an NIH award must include an acknowledgment of NIH award support and a disclaimer such as “Research reported in this publication was supported by the National Institute of Allergy and Infectious Diseases of the National Institutes of Health under Award Number U01AI096299 (PI: Anastos and Nash). The content is solely the responsibility of the authors and does not necessarily represent the official views of the National Institutes of Health.” Prior to issuing a press release concerning the outcome of this research, please notify the NIH awarding Institute/Center in advance to allow for coordination.

**Acknowledgements List**

**Site investigators and cohorts:**

Nimbona Pélagie, ANSS, Burundi; Patrick Gateretse, Jeanine Munezero, Valentin Nitereka, Théodore Niyongabo, Christelle Twizere, Centre National de Reference en Matiere de VIH/SIDA, Burundi; Hélène Bukuru, Thierry Nahimana, CHUK, Burundi; Jérémie Biziragusenyuka, Risase Scholastique Manyundo, HPRC, Burundi; Rogers Ajeh, Mark Benwi, Anastase Dzudie, Akindeh Mbuh, CRENC & Douala General Hospital, Cameroon; Kien Atsu, Tabeyang Mbuh, Bamenda Hospital, Cameroon; Djenabou Amadou, Eric Ngassam, Eric Walter Pefura Yone, Jamot Hospital, Cameroon; Alice Ndelle Ewanoge, Norbert Fuhngwa, Chris Moki, Limbe Regional Hospital, Cameroon; Catherine Akele, Faustin Kitetele, Patricia Lelo, Martine Tabala, Kalembelembe Pediatric Hospital, Democratic Republic of Congo; Emile Wemakoy Okitolonda, Landry Wenzi, Kinshasa School of Public Health, Democratic Republic of Congo; Merlin Diafouka, Martin Herbas Ekat, Dominique Mahambou Nsonde, CTA Brazzaville, Republic of Congo; Adolphe Mafou, CTA Pointe-Noire, Republic of Congo; Jean Claude Dusingize, Gallican Kubwimana, Pacifique Mugenzi, Benjamin Muhoza, Athanase Munyaneza, Emmanuel Ndahiro, Diane Nyiransabimana, Jean d'Amour Sinayobye, Vincent Sugira, Rwanda Military Hospital, Rwanda; Fidele Ntarambirwa, Bethsaida Hospital, Rwanda; Yvonne Tuyishimire, Busanza Health Center, Rwanda; Theogene Hakizimana, Gahanga Health Center, Rwanda; Josephine Ayinkamiye, Gikondo Health Center, Rwanda; Sandrine Mukantwali, Kabuga Health Center, Rwanda; Henriette Kayitesi, Olive Uwamahoro, Kicukuro Health Center, Rwanda; Viateur Habumuremyi, Nyarugunga Health Center, Rwanda; Joyce Mukamana, Masaka Health Center, Rwanda; Chantal Benekigeri, Gilbert Mbaraga, WE-ACTx Health Center, Rwanda.

**Coordinating and Data Centers:**

Adebola Adedimeji, Kathryn Anastos, Lynn Murchison, Jonathan Ross, Albert Einstein College of Medicine, USA; Diane Addison, Ellen Brazier, Heidi Jones, Elizabeth Kelvin, Sarah Kulkarni, Grace Liu, Denis Nash, Olga Tymejczyk, City University of New York (CUNY), USA; Batya Elul, Columbia University, USA; Xiatao Cai, Don Hoover, Chunshan Li, Qiuhu Shi, Data Solutions, USA; Marcel Yotebieng, Ohio State University, USA; Mark Kuniholm, University at Albany, State University of New York, USA; Andrew Edmonds, University of North Carolina at Chapel Hill, USA; Stephany Duda; Vanderbilt University School of Medicine, USA; April Kimmel, Virginia Commonwealth University School of Medicine, USA.

**NA-ACCORD**

**Funding acknowledgement**

The content is solely the responsibility of the authors and does not necessarily represent the official views of the National Institutes of Health. This work was supported by National Institutes of Health grants U01AI069918, F31AI124794, F31DA037788, G12MD007583, K01AI093197, K01AI131895, K23EY013707, K24AI065298, K24AI118591, K24DA000432, KL2TR000421, M01RR000052, N01CP01004, N02CP055504, N02CP91027, P30AI027757, P30AI027763, P30AI027767, P30AI036219, P30AI050410, P30AI094189, P30AI110527, P30MH62246, R01AA016893, R01CA165937, R01DA011602, R01DA012568, R01 AG053100, R24AI067039, U01AA013566, U01AA020790, U01AI031834, U01AI034989, U01AI034993, U01AI034994, U01AI035004, U01AI035039, U01AI035040, U01AI035041, U01AI035042, U01AI037613, U01AI037984, U01AI038855, U01AI038858, U01AI042590, U01AI068634, U01AI068636, U01AI069432, U01AI069434, U01AI103390, U01AI103397, U01AI103401, U01AI103408, U01DA03629, U01DA036935, U01HD032632, U10EY008057, U10EY008052, U10EY008067, U24AA020794,U54MD007587, UL1RR024131, UL1TR000004, UL1TR000083, UL1TR000454, UM1AI035043, Z01CP010214 and Z01CP010176; contracts CDC-200-2006-18797 and CDC-200-2015-63931 from the Centers for Disease Control and Prevention, USA; contract 90047713 from the Agency for Healthcare Research and Quality, USA; contract 90051652 from the Health Resources and Services Administration, USA; grants CBR-86906, CBR-94036, HCP-97105 and TGF-96118 from the Canadian Institutes of Health Research, Canada; Ontario Ministry of Health and Long Term Care; and the Government of Alberta, Canada. Additional support was provided by the National Cancer Institute, National Institute for Mental Health and National Institute on Drug Abuse.

**NA-ACCORD Collaborating Cohorts and Representatives**:

AIDS Clinical Trials Group Longitudinal Linked Randomized Trials: Constance A. Benson and Ronald J. Bosch; AIDS Link to the IntraVenous Experience: Gregory D. Kirk; Fenway Health HIV Cohort: Kenneth H. Mayer and Chris Grasso; HAART Observational Medical Evaluation and Research: Robert S. Hogg, P. Richard Harrigan, Julio SG Montaner, Benita Yip, Julia Zhu, Kate Salters and Karyn Gabler; HIV Outpatient Study: Kate Buchacz and Jun Li; HIV Research Network: Kelly A. Gebo and Richard D. Moore; Johns Hopkins HIV Clinical Cohort: Richard D. Moore; John T. Carey Special Immunology Unit Patient Care and Research Database, Case Western Reserve University: Benigno Rodriguez; Kaiser Permanente Mid-Atlantic States: Michael A. Horberg; Kaiser Permanente Northern California: Michael J. Silverberg; Longitudinal Study of Ocular Complications of AIDS: Jennifer E. Thorne; Multicenter Hemophilia Cohort Study–II: Charles Rabkin; Multicenter AIDS Cohort Study: Joseph B. Margolick, Lisa P. Jacobson and Gypsyamber D’Souza; Montreal Chest Institute Immunodeficiency Service Cohort: Marina B. Klein; Ontario HIV Treatment Network Cohort Study: Abigail Kroch, Ann Burchell, Beth Rachlis, Anita Rachlis, Patrick Cupido and Joanne Lindsay; Retrovirus Research Center, Bayamon Puerto Rico:  Robert F. Hunter-Mellado and Angel M. Mayor; Southern Alberta Clinic Cohort: M. John Gill; Study of the Consequences of the Protease Inhibitor Era: Steven G. Deeks and Jeffrey N. Martin; Study to Understand the Natural History of HIV/AIDS in the Era of Effective Therapy: Jun Li and John T. Brooks; University of Alabama at Birmingham 1917 Clinic Cohort: Michael S. Saag, Michael J. Mugavero and James Willig; University of California at San Diego: William C. Mathews; University of North Carolina at Chapel Hill HIV Clinic Cohort: Joseph J. Eron and Sonia Napravnik; University of Washington HIV Cohort: Mari M. Kitahata, Heidi M. Crane and Daniel R. Drozd; Vanderbilt Comprehensive Care Clinic HIV Cohort: Timothy R. Sterling, David Haas, Peter Rebeiro and Megan Turner; Veterans Aging Cohort Study: Amy C. Justice, Robert Dubrow, and David Fiellin; Women's Interagency HIV Study: Stephen J. Gange and Kathryn Anastos

**NA-ACCORD Study Administration**:
Executive Committee: Richard D. Moore, Michael S. Saag, Stephen J. Gange, Mari M. Kitahata, Keri N. Althoff, Michael A. Horberg, Marina B. Klein, Rosemary G. McKaig and Aimee M. Freeman; Administrative Core: Richard D. Moore and Aimee M. Freeman; Data Management Core: Mari M. Kitahata, Stephen E. Van Rompaey, Heidi M. Crane, Daniel R. Drozd, Liz Morton, Justin McReynolds and William B. Lober; Epidemiology and Biostatistics Core: Stephen J. Gange, Keri N. Althoff, Jennifer S. Lee, Bin You, Brenna Hogan, Jinbing Zhang, Jerry Jing, Elizabeth Humes, and Sally Coburn

**East Africa IeDEA**

**Funding acknowledgement-Adult and Pediatric**

Research reported in this publication was supported by the National Institute Of Allergy And Infectious Diseases (NIAID), Eunice Kennedy Shriver National Institute Of Child Health & Human Development (NICHD), National Institute On Drug Abuse (NIDA), National Cancer Institute (NCI), and the National Institute of Mental Health (NIMH), in accordance with the regulatory requirements of the National Institutes of Health under Award Number U01AI069911East Africa IeDEA Consortium. The content is solely the responsibility of the authors and does not necessarily represent the official views of the National Institutes of Health.

**Site investigators and cohorts (without data managers):**

Lameck Diero, Samuel Ayaya, MOI University, AMPATH Plus, Eldoret, Kenya; Elizabeth Bukusi, KEMRI (Kenya Medical Research Institute), Kisumu, Kenya; John Ssali, Masaka Regional Referral Hospital, Masaka, Uganda; Mwebesa Bosco Bwana, Mbarara University of Science and Technology (MUST), Mbarara, Uganda; Barbara Castelnuovo, Infectious Diseases Institute (IDI), Mulago, Uganda; Fred Nalugoda, Rakai Health Sciences Program, Kalisizo, Uganda; G.R. Somi, NACP (National AIDS Control Program) Dar es Salaam, Tanzania; Rita Elias Lyamuya, Morogoro Regional Hospital, Morogoro, Tanzania; Kapella Ngonyani, Tumbi Regional Hospital, Pwani, Tanzania; Mark Urassa, Denna Michael, National Institute for Medical Research (NIMR), Kisesa HDSS, Mwanza, Tanzania; Kara Wools-Kaloustian, Constantin Yiannoutsos, Rachel Vreeman, Indiana University School of Medicine, Indiana University, Indianapolis, IN, USA; Batya Elul, Columbia University, New York City, NY, USA; Jennifer Syvertsen, Ohio State University, Columbus, OH, USA; Rami Kantor, Brown University/Miriam Hospital, Providence, RI, USA; Jeffrey Martin, Craig Cohen, University of California, San Francisco, CA, USA; Paula Braitstein, University of Toronto, Toronto, Canada

**Site investigators and cohorts (with data managers):**

Diero L, Ayaya S, Sang E, MOI University, AMPATH Plus, Eldoret, Kenya; Bukusi E, Charles Karue Kibaara, Elisheba Mutegi, KEMRI (Kenya Medical Research Institute), Kisumu, Kenya; John Ssali, Mathew Ssemakadde, Masaka Regional Referral Hospital, Masaka, Uganda; Mwebesa Bosco Bwana, Michael Kanyesigye, Mbarara University of Science and Technology (MUST), Mbarara, Uganda; Barbara Castelnuovo; John Michael Matovu, Infectious Diseases Institute (IDI), Mulago, Uganda; Fred Nalugoda, Francis X. Wasswa, Rakai Health Sciences Program, Kalisizo, Uganda; G.R. Somi, Joseph Nondi, NACP (National AIDS Control Program) Dar es Salaam, Tanzania; Rita Elias Lyamuya, Francis Mayanga, Morogoro Regional Hospital, Morogoro, Tanzania; Kapella Ngonyani, Jerome Lwali, Tumbi Regional Hospital, Pwani, Tanzania; Mark Urassa, Denna Michael, Richard Machemba, National Institute for Medical Research (NIMR), Kisesa HDSS, Mwanza, Tanzania; Kara Wools-Kaloustian, Constantin Yiannoutsos, Rachel Vreeman, Beverly Musick, Indiana University School of Medicine, Indiana University, Indianapolis, IN, USA; Batya Elul, Columbia University, New York City, NY, USA; Jennifer Syvertsen, Ohio State University, Columbus, OH, USA; Rami Kantor, Brown University/Miriam Hospital, Providence, RI, USA; Jeffrey Martin, Megan Wenger, Craig Cohen, Jayne Kulzer, University of California, San Francisco, CA, USA; Paula Braitstein, University of Toronto, Toronto, Canada

**IeDEA Southern Africa**

**Funding acknowledgement**

Each publication, press release, or other document about research supported by an NIH award must include an acknowledgment of NIH award support and a disclaimer such as ***“Research reported in this publication was supported by the National Institute of Allergy and Infectious Diseases of the National Institutes of Health under Award Number U01AI069924. The content is solely the responsibility of the authors and does not necessarily represent the official views of the National Institutes of Health.”*** Prior to issuing a press release concerning the outcome of this research, please notify the NIH awarding IC in advance to allow for coordination.

**Site investigators and cohorts:**

Gary Maartens, Aid for AIDS, South Africa; Carolyn Bolton, Michael Vinikoor, Centre for Infectious Disease Research in Zambia (CIDRZ), Zambia; Monique van Lettow, Dignitas, Malawi; Robin Wood, Gugulethu ART Programme, South Africa; Nosisa Sipambo, Harriet Shezi Clinic, South Africa; Frank Tanser, Africa Centre for Health & Population Studies (Hlabisa), South Africa; Andrew Boulle, Khayelitsha ART Programme, South Africa; Geoffrey Fatti, Kheth’Impilo, South Africa; Sam Phiri, Lighthouse Clinic, Malawi; Cleophas Chimbetete, Newlands Clinic, Zimbabwe; Karl Technau, Rahima Moosa Mother and Child Hospital, South Africa; Brian Eley, Red Cross Children's Hospital, South Africa; Josephine Muhairwe, SolidarMed Lesotho; Juan Burgos-Soto, SolidarMed Mozambique; Cordelia Kunzekwenyika, SolidarMed Zimbabwe, Matthew P Fox, Themba Lethu Clinic, South Africa; Hans Prozesky, Tygerberg Academic Hospital, South Africa.

**Data centers:**

Nina Anderegg, Marie Ballif, Lina Bartels, Julia Bohlius, Frédérique Chammartin, Benedikt Christ, Cam Ha Dao Ostinelli, Matthias Egger, Lukas Fenner, Per von Groote, Andreas Haas, Taghavi Katayoun, Eliane Rohner, Lilian Smith, Adrian Spörri, Gilles Wandeler, Elizabeth Zaniewski, Kathrin Zürcher, Institute of Social and Preventive Medicine, University of Bern, Switzerland; Andrew Boulle, Morna Cornell, Mary-Ann Davies, Victoria Iyun, Leigh Johnson, Mmamapudi Kubjane, Nicola Maxwell, Tshabakwane Nembandona, Patience Nyakato, Ernest Mokotoane, Gem Patten, Michael Schomaker, Priscilla Tsondai, Renee de Waal, School of Public Health and Family Medicine, University of Cape Town, South Africa.

**West Africa**

**Funding acknowledgement**

Each publication, press release, or other document about research supported by an NIH award must include an acknowledgment of NIH award support and a disclaimer such as “Research reported in this publication was supported by the US National Institutes of Health (NIAID, NICHD, NCI and NIMH) under Award Number U01AI069919 (PI: Dabis). The content is solely the responsibility of the authors and does not necessarily represent the official views of the National Institutes of Health.” Prior to issuing a press release concerning the outcome of this research, please notify the NIH awarding Institute/Centre in advance to allow for coordination.

**Site investigators and cohorts:**

Adult cohorts: Marcel Djimon Zannou, CNHU, Cotonou, Benin; Armel Poda, CHU Souro Sanou, Bobo Dioulasso, Burkina Faso; Fred Stephen Sarfo & Komfo Anokeye Teaching Hospital, Kumasi, Ghana; Eugene Messou, ACONDA CePReF, Abidjan, Cote d’Ivoire; Henri Chenal, CIRBA, Abidjan, Cote d’Ivoire; Kla Albert Minga, CNTS, Abidjan, Cote d’Ivoire; Emmanuel Bissagnene, & Aristophane Tanon, CHU Treichville, Cote d’Ivoire; Moussa Seydi, CHU de Fann, Dakar, Senegal; Akessiwe Akouda Patassi, CHU Sylvanus Olympio, Lomé, Togo.

Pediatric cohorts: Sikiratou Adouni Koumakpai-Adeothy,_CNHU, Cotonou, Benin; Lorna Awo Renner, Korle Bu Hospital, Accra, Ghana; Sylvie Marie N’Gbeche, ACONDA CePReF, Abidjan, Ivory Coast; Clarisse Amani Bosse, ACONDA_MTCT+, Abidjan, Ivory Coast; Kouadio Kouakou, CIRBA, Abidjan, Cote d’Ivoire; Madeleine Amorissani Folquet, CHU de Cocody, Abidjan, Cote d’Ivoire; François Tanoh Eboua, CHU de Yopougon, Abidjan, Cote d’Ivoire; Fatoumata Dicko Traore, Hopital Gabriel Toure, Bamako, Mali; Elom Takassi, CHU Sylvanus Olympio, Lomé,Togo

**Coordinating & data centers:**

François Dabis, Elise Arrive, Eric Balestre, Renaud Becquet, Charlotte Bernard, Shino Chassagne Arikawa, Alexandra Doring, Antoine Jaquet, Karen Malateste, Elodie Rabourdin, Thierry Tiendrebeogo, ADERA, Isped & INSERM U1219, Bordeaux, France.

Sophie Desmonde, Julie Jesson, Valeriane Leroy, Inserm 1027, Toulouse, France

Didier Koumavi Ekouevi, Jean-Claude Azani, Patrick Coffie, Abdoulaye Cissé, Guy Gnepa, Apollinaire Horo, Christian Kouadio, Boris Tchounga, PACCI, CHU Treichville, Abidjan, Côte d’Ivoire
