# Supplementary material for: Changes in rapid HIV treatment initiation after national “treat all” policy adoption in 6 sub-Saharan African countries: Regression discontinuity analysis
Source: PLoS Med. 2019 Jun 10;16(6):e1002822. doi: 10.1371/journal.pmed.1002822 (PMC6557472; doi:10.1371/journal.pmed.1002822)
Supplement: S2 Text — (DOCX) [file pmed.1002822.s007.docx]

**Supplement 1: Analysis concept sheet**

**Rapid HIV treatment initiation after Treat All adoption in six sub-Saharan African countries: regression discontinuity analysis.**

Olga Tymejczyk MPH, Ellen Brazier MA, Constantin T. Yiannoutsos PhD, Michael Vinikoor MD, Monique van Lettow PhD, Fred Nalugoda PhD, Mark Urassa MSc, Jean d’Amour Sinayobye MD, Peter F. Rebeiro PhD, Kara Wools-Kaloustian MD, Mary-Ann Davies PhD, Elizabeth Zaniewski MSc, Nanina Anderegg MSc, Grace Liu BA, Nathan Ford PhD, and Denis Nash PhD for the IeDEA Consortium

This concept sheet outlines the background, rationale, and early analytic plans for the present manuscript, and was approved by the IeDEA Executive Committee on May 3, 2018.

The concept outlines the following aspects of the present analysis:

1. Inclusion and exclusion criteria
2. Use of regression discontinuity analysis to assess ART initiation after country-level introduction of Treat All policies
3. Use of meta-analysis to obtain pooled estimates from country-level regression discontinuity analyses
4. Definition of ART initiation

The key differences between this proposal and the final analysis in the manuscript are:

1. The concept originally proposed to look at “timely” ART initiation, defined as within 3 months of enrollment. However, prior to analysis, we instead chose to assess “rapid” ART initiation, defined as within 30 days of enrollment. There were three reasons behind this change:
   1. A shorter-term outcome was more appropriate for the proposed regression discontinuity design, minimizing overlap in the outcome estimation windows between pre- and post-Treat All periods. With an outcome definition stretching out 3 months into the future, we would have had to exclude from analysis all patients who enrolled within 3 months before Treat All introduction, as their outcome could have been affected by the new policy. By looking at “rapid” ART initiation within 30 days of enrollment, we only had to exclude persons enrolling in the 30-day period prior to policy introduction to avoid this problem.
   2. Because we only had 6 months of follow-up data under Treat All from Malawi and 9 months from Zambia, the 30-day outcome definition allowed us to include more patients in the analysis. Using the 3-month definition would have required that we exclude patients who enrolled less than 3 months between the database close date, lowering the N available for analysis and shortening the analyzed Treat All period by 2 months.
2. We limited the scope of the regression discontinuity analysis to focus on expansion to Treat All instead of applying the method to all HIV treatment eligibility expansions, as we found the assessment of multiple expansions, across different points in time, to be too extensive to adequately address in a single paper. In addition, because countries differed in many other ART eligibility criteria that may not be completely documented in IeDEA patient data, even as they adopted the same CD4 count criteria, cross-country comparisons of prior expansions would have been extremely complex and with patients’ treatment eligibility status subject to possible misclassification. Although we did not apply the regression discontinuity analysis to prior treatment eligibility expansions, to provide rich context to the reader, we report descriptive statistics on periods before and after ART eligibility expansions to CD4 ≤350 and ≤500 cells/µl, describe the change in outcome across these periods, and present longer trends in rapid ART visually in a figure.
3. We did not do descriptive analyses of ART initiation levels out to 24 months after enrollment, as this was not informative after we limited the scope of analysis to changes in ART initiation after Treat All introduction (a great majority of patients in the analysis initiated treatment in the first 30 days under Treat All).
4. Regression discontinuity analysis was applied to individual patient-level data, not aggregate data (monthly proportion of rapid ART initiators).
5. ART initiation rates were not stratified by country income status because there was little heterogeneity in the country income status of the sites meeting our inclusion criteria for this analysis.
6. We did not conduct the proposed sensitivity analysis using Treat All adoption dates reported by sites in a recent IeDEA site survey because many sites could not report a specific month of site-level introduction. In addition, we were concerned that shifting the Treat All threshold dates to the median timing of site-level introduction would no longer estimate the effect of national adoption of Treat All policies. In contrast, using the dates of national policy adoption is a more conservative approach, which would bias results towards the null in situations of delayed policy implementation.
7. The multivariable analysis of factors associated with not initiating ART rapidly under Treat All had not been pre-specified in this concept. The team decided to explore this question after limiting the scope of the analysis to focus on the expansion to Treat All, as opposed to multiple eligibility expansions.

**Concept Proposal Submitted to and Approved by the IeDEA Executive Committee:**

| **Abstract:** (approximately 200 words) | **Background**: Despite improvements in access to ART, many HIV-infected people experience pre-treatment delays and/or large gaps in continuity of care prior to ART initiation. This results in mortality prior to ART initiation, substantial early mortality on ART, slower CD4 cell count response to ART, more complicated and costly clinical management, and missed opportunities to prevent HIV transmission.    **Objectives:**   1. Assess the influence of changes in national treatment guidelines on the proportion of patients initiating ART at the original site of enrollment, among adults enrolling in HIV care at IeDEA sites between 2004-2017.   **Methods**: The proportion of patients initiating ART at timepoints out to 24 months after enrollment will be calculated. Timely ART initiation will be defined as starting treatment within 3 months of enrollment. Regression discontinuity analysis of individual-level data will be used to assess country-level changes in timely ART initiation, aggregated across sites, after national ART eligibility expansions. Meta-analytic methods will be used to obtain pooled estimates of country-level changes in timely ART initiation after national guideline expansions. Analyses will be stratified by key patient demographic and clinical characteristics, as well as country income level. Estimates of timely ART initiation will also be stratified across four national ART eligibility guideline periods (CD4≤200, ≤350, ≤500, and universal test and treat (UTT)). |
| --- | --- |
| **Project outline:** (approximately 1000 words) | **Background**  Despite improvements in access to ART across the world (1) and increases in the median CD4 cell counts of adults initiating treatment,(2) many HIV-infected people in areas with the greatest burden of HIV continue to experience delays in treatment initiation and start ART with advanced HIV infection.(3-5) In 2012, among persons eligible for treatment in low- and middle-income countries under WHO’s 2010 ART guidelines, only 61% were receiving ART, and in 2013 when WHO expanded treatment eligibility criteria, only 34% of eligible patients were receiving ART.(1) WHO recommended universal testing and treatment for all people living with HIV in September 2015, however, adult ART coverage was only 46% worldwide at the end of 2015.(6)  Delays in HIV diagnosis and enrollment into HIV care, along with gaps in the continuity of care prior to ART initiation can result in mortality prior to ART initiation, substantial early mortality on ART,(7) slower CD4 cell count recovery,(8) more complicated and costly clinical management,(9) and missed opportunities to prevent onward HIV transmission.(10) Even in the era of universal ART eligibility, delays in ART initiation may still result from delayed HIV diagnosis, delayed enrollment in care, or delayed initiation of ART (once enrolled in HIV care).(5)  Delays across the HIV care continuum may be influenced by patient-, program-, and policy-level factors.(5) A variety of clinical and sociodemographic factors have been linked to late presentation to care(11) and late treatment initiation(12-14), including patient’s sex, pregnancy status, age, education, family composition, tuberculosis diagnosis, gaps in pre-ART care, treatment eligibility at the time of presentation to care, and source of referral to HIV care.(12, 15-20) The timing of ART initiation is also greatly influenced by changes in national HIV treatment guidelines.(14, 21) Data from the IeDEA collaboration can be used to better understand the extent of delays in ART initiation among various groups of patients retained in HIV care and to inform strategies for achieving the UNAIDS goal of 90% ART coverage by 2020 (part of the 90-90-90 targets).(22) IeDEA data can also be used to examine the extent to which changing treatment guidelines in each country influence key outcomes, such as the proportion initiating ART at the original site. This analysis aims to assess whether ART guideline expansions, which progressively eliminated eligibility barriers to treatment, were accompanied by increases in timely ART initiation.  **Primary objective**   1. Assess the influence of changes in national treatment guidelines on the proportion of patients initiating ART at the original site of enrollment.   We will stratify estimates by:   - Sex - Age at enrollment in HIV care - CD4 cell count at enrollment in HIV care - World Bank country income group - IeDEA region and country - Enrollment period, mapped to country-level ART eligibility guidelines in effect at the time (CD4≤200, ≤350, ≤500, and UTT).   **Eligibility criteria**  All adult IeDEA sites that systematically record data for patients before ART initiation are eligible for inclusion. All patients enrolling in HIV care in 2004 or later at 16 years of age or older, regardless of whether they eventually initiated ART, are eligible for inclusion in these analyses.  **Exclusion criteria**   - Patients known not to be ART naïve at enrollment - Patients who transferred into the site regardless of ART status at the time of transfer - Sites with no pre-ART data available   **Primary outcomes**   - ART initiation: start of a regimen of at least three antiretroviral drugs, in accordance with the IeDEA/WHO collaboration definition. - Timely ART initiation: ART initiation within 3 months of enrollment in HIV care   **Other definitions**   - CD4 cell count at enrollment in HIV care: CD4 cell count measurement closest to the date of HIV care enrollment within 3 months after enrollment date and no later than 1 week after ART initiation.   **Statistical methods**  Proportion of patients initiating ART at timepoints out to 24 months after enrollment will be calculated and graphed as curves stratified by enrollment periods.  Unlike prior analyses led by this team under this concept, ART incidence will no longer be estimated via competing risk methods, because the generally short follow-up time available under largely recently introduced UTT guidelines does not allow sufficient time to implement a loss-to-follow-up definition. Further, the main outcome of interest in this analysis, ART initiation by 3 months after enrollment, is short-term, limiting the potential relevance of competing events such as death, which are not expected to be recorded for an appreciable number of patients in the analysis by this timepoint. Additionally, by no longer requiring 12 months of possible follow-up between enrollment in care and database close date (which was previously the case due to our definition of loss to follow-up), we will be able to include more patients in the analysis.  In addition, unlike prior analyses under this concept, regression discontinuity analysis will be used to assess country-level changes in timely ART initiation after national ART eligibility expansions, in the subset of countries with at least 10 months of follow-up data under UTT. Country-level proportion of patients initiating ART by 3 months after enrollment will be calculated *for each month* of available data (resulting in a minimum of 6 single-month estimates under UTT, due to the minimum requirement of 10 months of data under UTT). For example, for patients who enrolled in care between January 1^st^ and January 31^st^, the outcome would be calculated using data out to May 1^st^, giving patients enrolled on the last day of January 3 months (or 90 days) to initiate ART:  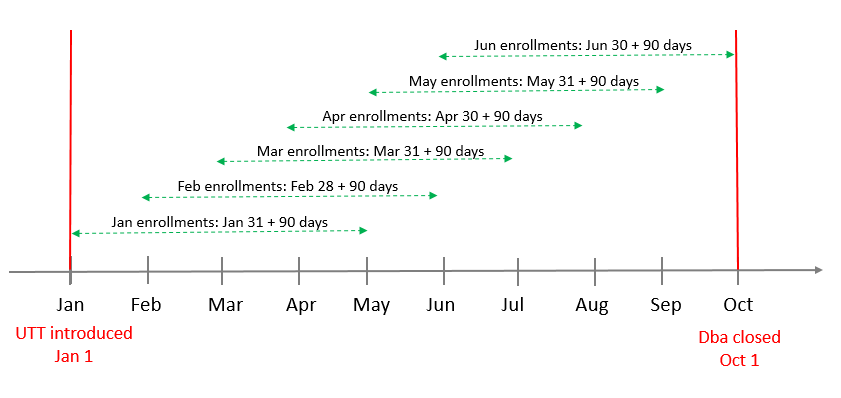  In each country, 4 sets of intercept & slope coefficients will be estimated for each guideline period, with 3 discontinuities in the model (at the time of guideline expansion to CD4≤350, ≤500, and UTT, respectively).   - For countries with only 3 distinct guideline periods (e.g., countries that expanded treatment eligibility from CD4≤350 to UTT, directly), models will be adapted to include only 2 discontinuities. - For countries with low numbers of new enrollments per month, data may be aggregated over 2-month periods instead of 1 month.   For each of the 3 guideline expansions, meta-analytic methods will be used to obtain pooled estimates of country-level changes in model intercepts and slopes after guideline expansions.  Estimates of timely ART initiation will be stratified by key patient demographic and clinical characteristics, country income level, and the period of enrollment in HIV care, mapped to 4 national ART eligibility guideline periods (CD4≤200, ≤350, ≤500, and UTT). Regression discontinuity analyses will not be stratified by these variables, as the underlying aggregation of data by country and single month of enrollment will likely preclude further stratification due to low Ns. Stratification by enrollment CD4 count would be particularly difficult due to the declining proportion of patients with enrollment CD4 counts available, which we have documented in prior analyses under this concept.  Sensitivity analyses will be completed to correct estimates of timely ART initiation for possible lags between national ART expansion policy adoption and site-level implementation, For this purpose, timely ART initiation under UTT will be recalculated with the start of the UTT period defined not by national policy adoption date, but by median date of site-level implementation, as reported for each country by IeDEA sites that participated in the 2017 IeDEA general site assessment (SA3.0).  **Sample size considerations**  This study will include all available data from patients with 16 years of age or older enrolled in HIV care at eligible sites. No power calculations have been performed. |
| **References:** | 1. Joint United Nations Programme on HIV/AIDS. Global Report: UNAIDS Report on the Global AIDS Epidemic 2013. Geneva: UNAIDS, 2013.  2. The IeDEA and ART Cohort Collaborations. Immunodeficiency at the start of combination antiretroviral therapy in low-, middle- and high-income countries. Journal of acquired immune deficiency syndromes (1999). 2014;65(1):e8-e16.  3. Avila D, Althoff KN, Mugglin C, Wools-Kaloustian K, Koller M, Dabis F, et al. Immunodeficiency at the start of combination antiretroviral therapy in low-, middle-, and high-income countries. Journal of acquired immune deficiency syndromes. 2014;65(1):e8-16.  4. Pati R, Lahuerta M, Elul B, Okamura M, Alvim MF, Schackman B, et al. Factors associated with loss to clinic among HIV patients not yet known to be eligible for antiretroviral therapy (ART) in Mozambique. J Int AIDS Soc. 2013;16:18490.  5. Lahuerta M, Ue F, Hoffman S, Elul B, Kulkarni SG, Wu Y, et al. The Problem of Late ART Initiation in Sub-Saharan Africa: A Transient Aspect of Scale-up or a Long-term Phenomenon? J Health Care Poor Underserved. 2013;24(1):359-83.  6. Joint United Nations Programme on HIV/AIDS. Global AIDS Update 2016. 2016.  7. Lawn SD, Harries AD, Anglaret X, Myer L, Wood R. Early mortality among adults accessing antiretroviral treatment programmes in sub-Saharan Africa. AIDS. 2008;22(15):1897-908.  8. Nash D, Katyal M, Brinkhof MW, Keiser O, May M, Hughes R, et al. Long-term immunologic response to antiretroviral therapy in low-income countries: a collaborative analysis of prospective studies. AIDS (London, England). 2008;22(17):2291-302.  9. Krentz HB, Auld MC, Gill MJ. The high cost of medical care for patients who present late (CD4 <200 cells/microL) with HIV infection. HIV medicine. 2004;5(2):93-8.  10. Cohen MS, Chen YQ, McCauley M, Gamble T, Hosseinipour MC, Kumarasamy N, et al. Prevention of HIV-1 infection with early antiretroviral therapy. N Engl J Med. 2012;365(6):493-505.  11. Hoffman S, Wu Y, Lahuerta M, Kulkarni SG, Nuwagaba-Biribonwoha H, Sadr WE, et al. Advanced disease at enrollment in HIV care in four sub-Saharan African countries: change from 2006 to 2011 and multilevel predictors in 2011. AIDS. 2014;28(16):2429-38.  12. Lahuerta M, Wu Y, Hoffman S, Elul B, Kulkarni SG, Remien RH, et al. Advanced HIV Disease at Entry into HIV Care and Initiation of Antiretroviral Therapy During 2006-2011: Findings From Four Sub-Saharan African Countries. Clinical infectious diseases : an official publication of the Infectious Diseases Society of America. 2014;58(3):432-41.  13. Mutimura E, Addison D, Anastos K, Hoover D, Dusingize JC, Karenzie B, et al. Trends in and correlates of CD4+ cell count at antiretroviral therapy initiation after changes in national ART guidelines in Rwanda. AIDS (London, England). 2015;29(1):67-76.  14. Nash D, Tymejczyk O, Gadisa T, Kulkarni SG, Hoffman S, Yigzaw M, et al. Factors associated with initiation of antiretroviral therapy in the advanced stages of HIV infection in six Ethiopian HIV clinics, 2012 to 2013. J Int AIDS Soc. 2016;19(1):20637.  15. Abaynew Y, Deribew A, Deribe K. Factors associated with late presentation to HIV/AIDS care in South Wollo ZoneEthiopia: a case-control study. AIDS research and therapy. 2011;8:8.  16. Drain PK, Losina E, Parker G, Giddy J, Ross D, Katz JN, et al. Risk factors for late-stage HIV disease presentation at initial HIV diagnosis in Durban, South Africa. PloS one. 2013;8(1):e55305.  17. Geng EH, Hunt PW, Diero LO, Kimaiyo S, Somi GR, Okong P, et al. Trends in the clinical characteristics of HIV-infected patients initiating antiretroviral therapy in Kenya, Uganda and Tanzania between 2002 and 2009. Journal of the International AIDS Society. 2011;14:46.  18. Hoffman S, Wu Y, Lahuerta M, Kulkarni SG, Nuwagaba-Biribonwoha H, Sadr WE, et al. Advanced disease at enrollment in HIV care in four sub-Saharan African countries: change from 2006 to 2011 and multilevel predictors in 2011. AIDS (London, England). 2014;28(16):2429-38.  19. Mulissa Z, Jerene D, Lindtjorn B. Patients present earlier and survival has improved, but pre-ART attrition is high in a six-year HIV cohort data from Ethiopia. PloS one. 2010;5(10):e13268.  20. Zango A, Dube K, Kelbert S, Meque I, Cumbe F, Chen PL, et al. Determinants of Prevalent HIV Infection and Late HIV Diagnosis among Young Women with Two or More Sexual Partners in Beira, Mozambique. PloS one. 2013;8(5):e63427.  21. Mutimura E, Addison D, Anastos K, Hoover DR, Dusingize JC, Karenzi B, et al. Trends in and Determinants of CD4+ Cell Count at ART Initiation after Changes in National ART Guidelines in Rwanda. AIDS. 2015;29(1):67-76.  22. Joint United Nations Programme on HIV/AIDS. 90-90-90: an ambitious treatment target to help end the AIDS epidemic. Report Geneva: UNAIDS. 2014. |
| **Ethics:** | *Select as appropriate:*  This concept uses only the IeDEA standard dataset and is covered by the core IeDEA ethics approvals.  This concept requires additional collection of health-related data, measurements or tests, or sampling of biological material not included in the IeDEA standard dataset. Additional ethics approval is required. |

Bottom of Form
